# Incidence, hospitalization, and mortality in children aged 5 years and younger with respiratory syncytial virus‐related diseases: A systematic review and meta‐analysis

**DOI:** 10.1111/irv.13145

**Published:** 2023-05-22

**Authors:** Yuping Duan, Mingyue Jiang, Qiangru Huang, Mengmeng Jia, Weizhong Yang, Luzhao Feng

**Affiliations:** ^1^ School of Population Medicine and Public Health Chinese Academy of Medical Sciences and Peking Union Medical College Beijing China

**Keywords:** burden of disease, children, meta‐analysis, respiratory syncytial virus, surveillance system, systematic review

## Abstract

**Objectives:**

Respiratory syncytial virus (RSV) is a leading cause of lower respiratory tract infection in young children. We aimed to analyze the factors affecting the estimation of RSV‐related disease burden, and to provide evidence to help establish a surveillance system.

**Methods:**

We searched the English‐ and Chinese‐language databases for articles published between January 1, 2010 and June 2, 2022. The quality of the included articles was assessed using the Agency for Healthcare Research and Quality scale. Random‐effects models were used for data synthesis and subgroup analyses. This review was registered in the Prospective Register of Systematic Reviews (PROSPERO: CRD42022372972).

**Results:**

We included 44 studies (149,321,171 participants), all of which were of medium or high quality. The pooled RSV‐related disease incidence, hospitalization rate, in‐hospital mortality, and overall mortality rates in children aged 5 years and younger were 9.0 per 100 children per year (95% confidence interval [CI]: 7.0–11.0), 1.7 per 100 children per year (95% CI: 1.3–2.1), 0.5 per 100 children per year (95% CI: 0.4–0.5), and 0.05 per 100 children per year (95% CI: 0.04–0.06), respectively. Age, economics, surveillance types, case definition, and data source were all recognized as influencing factors.

**Conclusions:**

A standardized and unified RSV surveillance system is required. Case definition and surveillance types should be fully considered for surveillance of different age groups.

## 1 INTRODUCTION

Respiratory syncytial virus (RSV) is a ubiquitous respiratory virus with a single‐stranded negative‐sense RNA.^1^ RSV is one of the major causes of acute lower respiratory tract infection (ALRI) in children aged under 5 years, with a population attribution fraction of 18.2% (95% confidence interval [CI]: 17.4–19.0%) in developing countries.^2^ Further, ALRI is a leading cause of death globally in children under 5 years.^3^ Early childhood RSV‐related ALRI may be associated with subsequent development of recurrent wheezing and asthma,^4^ which may reduce with quality of life, and impose a considerable burden on healthcare utilization.^5^

Estimates have been published of the global disease burden of RSV ALRI in children under 5 years of age in 2005, 2015, and 2019.^6^, ^7^, ^8^ Factors affecting the RSV disease burden vary according to age, region, type of surveillance,^8^ case definition,^9^ and the diagnostic tests.^10^ Some factors may lead to an underestimation of the burden of RSV‐related disease.^11^ Moreover, due to heterogeneity among studies, the results of different studies lack comparability. Therefore, it is important to identify the factors that influence estimates of RSV burden in order to develop a standardized RSV surveillance system.^12^

In recent years, the epidemiology of RSV has changed owing to the public health and social measures implemented during the coronavirus disease 2019 (COVID‐19) pandemic.^13^, ^14^ RSV incidence, out‐of‐season activity, and health‐service demands may increase when these preventive measures are relaxed.^14^ The potential effect of COVID‐19 and RSV coinfection is a concern, especially in infants who have not been vaccinated during the COVID‐19 pandemic.^14^ Considerable progress has been made regarding RSV prevention, including thorough research into maternal vaccination in the antenatal period, and administration of long‐acting monoclonal antibodies to infants;^15^ thus, exploring key factors for establishing a standardized surveillance system will further provide evidence for the development of the national vaccination program. To achieve these objectives, we performed a systematic literature review and meta‐analysis to clarify the RSV‐related disease incidence, hospitalization rate, in‐hospital mortality rate, and overall mortality among children aged 5 years and younger. We aimed to explore factors that may impact RSV‐related disease burden estimates and help make suggestions for a standard and feasible surveillance system.

## 2 MATERIALS AND METHODS

### 2.1 Search strategy and selection criteria

This study was carried out according to the Preferred Reporting Items for Systematic Review and Meta‐Analysis (PRISMA) guidelines,^16^ and the protocol was registered in the Prospective Register of Systematic Reviews (PROSPERO, CRD42022372972). We performed a systematic literature review of peer‐reviewed articles published in English or Chinese, and searched English‐ and Chinese‐language databases, including PubMed, Embase, The Cochrane Library, Chinese National Knowledge Infrastructure, Wanfang Database, Chinese Biomedical Literature Database, and Chinese Scientific Journal Database, for articles published from January 1, 2010 to June 2, 2022. Medical Subject Headings terms and free text words were combined to conduct a literature search. For each database, combinations of the following search terms were used: “respiratory syncytial virus infections,” “respiratory syncytial viruses,” “respiratory syncytial virus, human,” “child,” “preschool,” “infant,” “pediatrics,” “schools, nursery,” “nurseries, infant,” “nurseries, hospital,” “incidence,” “morbidity,” “prevalence,” “inpatients,” “hospitalization,” and “mortality.” The search strategy is shown in Table S1.

After removing duplicate articles, the titles and/or abstracts were screened by two independent reviewers to meet the following criteria: (1) the included children were aged ≤5 years, who had RSV and were otherwise healthy; (2) the outcome indicators were RSV‐related disease incidence, hospitalization rate, in‐hospital mortality rate, and overall mortality; (3) the study design was cohort, cross‐sectional, case‐control, case‐cohort, or nested case‐control; and (4) the sample size was over 1000. We excluded studies without data on children aged under 5 years or that only reported data on children with comorbidities or underlying conditions, such as Down syndrome, congenital heart disease, and immunodeficiency. Further, we excluded studies that reported proportions, comparison of RSV diagnostic tests, animal research, repeated publications, letters, comments, case reports, case series, editorials, reviews, pure modeling studies or modeling studies for trend estimation, and studies with unavailable full text or those that were published in languages other than Chinese or English. The full texts of included studies were reviewed by two independent reviewers to assess their eligibility based on the inclusion and exclusion criteria. A third reviewer was consulted to reach consensus if the two reviewers' assessments did not concur.

### 2.2 Data extraction and quality assessment

Data on the eligible studies were extracted by two researchers independently and simultaneously, and then exchanged for checking to prevent data errors. When data were inconsistent among reviewers, they discussed and reassessed the article until they reached agreement. The data extraction checklist included the name of the first author, survey period, region or country where the study was conducted, race, study design (active or passive surveillance), data source (national database or hospital database), case definition (extended acute respiratory illness [ARI] or influenza‐like illness [ILI], Table S2), sample collection or detection method, sample size, age group, sex, quality score, and main outcomes that included RSV‐related disease incidence, hospitalization rate, in‐hospital mortality rate, and overall mortality (Tables S3–S6).

Two independent reviewers used the Agency for Healthcare Research and Quality assessment tool (Table S7) to evaluate the risk of bias for cross‐sectional studies.^17^

### 2.3 Data synthesis and analysis

Pooled estimates of one‐group meta‐analyses were used to estimate the synthesis effect size and 95% CIs for RSV‐related disease incidence, hospitalization rate, in‐hospital mortality rate, and overall mortality in children aged 0–5 years. Subgroup analyses were performed to explore potential determinants affecting the RSV‐related disease burden, such as age, income level (according to the 2021 World Bank classification), surveillance type (active or passive surveillance), case definition (extended ARI or ILI), and data source. Table S2 contains detailed definitions and criteria for subgroup division. The Cochrane Q test and *I* ^2^ were used to assess the heterogeneity of the studies, with <25%, 25%–50%, and >50% indicating low‐, moderate‐, and high‐level heterogeneity, respectively. As the heterogeneity of all pooled results was greater than 50%, a random‐effects model was used for the analysis, and the pooled effect size was weighted by the random‐effects model. Forest plots were used to present the data. Sensitivity analyses were performed by omitting each study individually to determine whether any included studies had a marked impact on the results and to examine the robustness of the overall effect. Potential publication bias was evaluated graphically and quantitatively using funnel plots and Egger's test, respectively. (*p‐*Values <0.05 indicated significant publication bias.) All statistical analyses were performed using Stata 17.0 (Stata Corp; College Station, TX, USA).

## 3 RESULTS

### 3.1 Search outcomes

**FIGURE 1:**
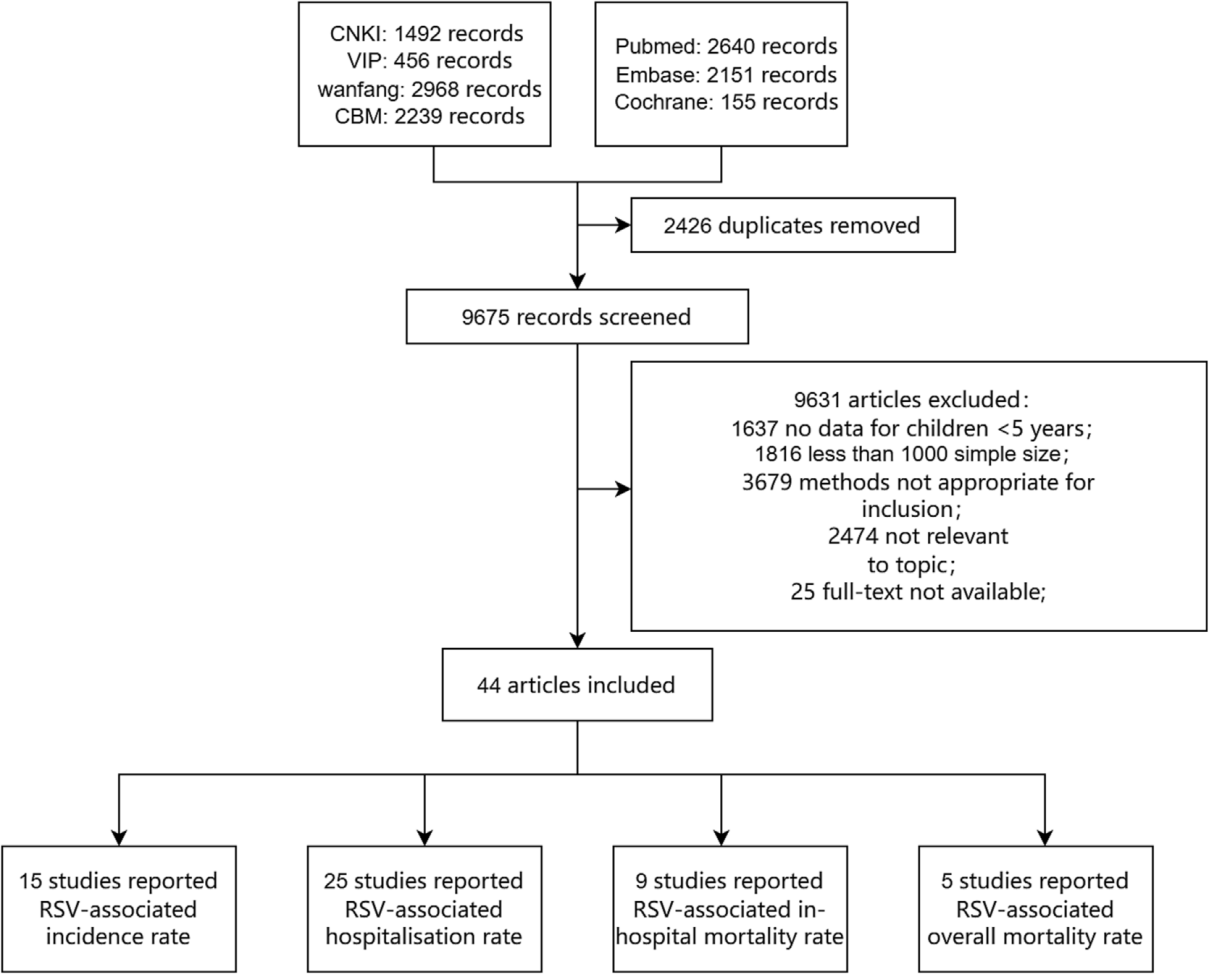
Flow diagram for selection of studies. CBM, Chinese biomedical literature database; CNKI, Chinese National Knowledge Infrastructure; RSV, respiratory syncytial virus; VIP, Chinese Scientific Journal Database.

A total of 12,101 records were retrieved, and 2426 duplicates were excluded. Subsequently, an additional 9675 records were excluded after reviewing the title and abstract. A total of 44 studies were included in the final meta‐analysis, of which 34 were classified as high quality, and 10 were classified as moderate quality (Table S7). The detailed selection process is shown in Figure 1.

### 3.2 Characteristics of the studies

A total of 149,321,171 participants took part in the 44 included studies, with study sample sizes ranging from 1153 to 30,417,106. The first study was conducted in 1997 and published in 2015, whereas the most recent study was conducted in 2020 and published in 2022. Fifteen of the included studies reported RSV‐related disease incidence in 107,527 participants, 25 reported RSV‐related hospitalization rates in 62,691,095 participants, nine reported RSV‐related in‐hospital mortality rates in 55,149,680 participants, and five reported RSV‐related overall mortality in 31,317,106 participants. Eleven surveys (25.0%) employed active surveillance, 31 (70.5%) employed passive surveillance, and two studies (4.5%) did not mention the surveillance type. Twenty‐seven studies (61.4%) were based on national databases, 13 (29.5%) were based on sentinel hospital databases, and four (9.1%) were birth‐cohort studies. Two studies (4.5%) used the ILI definition for patient inclusion, and the remaining (95.5%) used the extended ARI definition. Eighteen studies (40.9%) were conducted in high‐income countries, 23 (52.3%) in middle‐income countries, and three (6.8%) in low‐income countries. Overall, participants were aged 0–5 years. A summary of the characteristics of the included studies is shown in Tables S3–S6.

### 3.3 Synthesis of results

**FIGURE 2:**
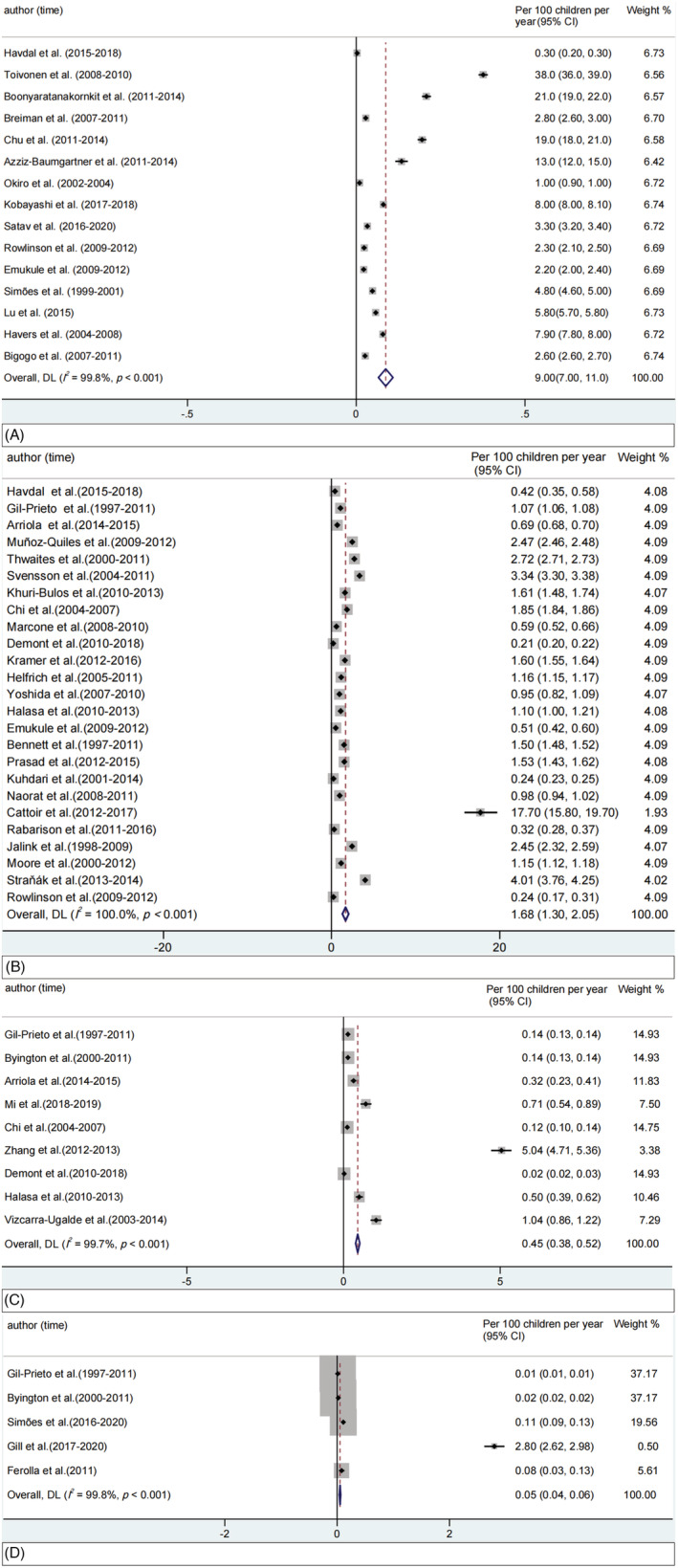
Synthesis of RSV‐associated incidence, hospitalization rate, in‐hospital mortality rate and overall mortality rate among children aged 5 years and younger. (A) RSV‐related incidence; (B) RSV‐related hospitalization rate; (C) RSV‐related in‐hospital mortality rate; (D) RSV‐related overall mortality rate. CI, confidential interval; RSV, respiratory syncytial virus; weights are from random‐effects model.

Based on the random‐effects model, pooled incidence, hospitalization rate, in‐hospital mortality rate, and overall mortality rate among children aged 5 years and younger were 9.0 (95% CI: 7.0–11.0), 1.7 (95% CI: 1.3–2.1), 0.5 (95% CI: 0.4–0.5), and 0.05 per 100 children per year (95% CI: 0.04–0.06), respectively (Figure 2).

### 3.4 Subgroup analysis

As shown in Table 1 and Figures S1–S4, subgroup analyses of RSV‐related disease incidence, hospitalization rate, in‐hospital mortality rate, and overall mortality rate varied according to age, income level, type of surveillance, case definition, and the data source.

**TABLE 1:** Subgroup analysis of RSV‐related incidence, hospitalization rate, in‐hospital mortality rate, and overall mortality rate among children aged 5 years and younger.

Subgroups	Per 100 children per year (95% CI)	*I* ^*2*^(%)	*p*‐Value	Number of included studies
Ages				
Incidence rate				
0–1 years old^a^	9.0 (6.0–12.0)	99.4	<0.001	9
0–2 years old^a^	9.0 (7.0–11.0)	99.8	<0.001	9
2–5 years old^a^	3.0 (2.0–5.0)	96.0	<0.001	5
Hospitalization rate				
0–1 years old	1.8 (1.3–2.3)	100.0	<0.001	14
0–2 years old	1.2 (0.5–1.9)	100.0	<0.001	11
2–5 years old	0.3 (0.2–0.4)	99.7	<0.001	8
In‐hospital mortality rate				
0–1 years old	1.1 (0.9–1.4)	99.7	<0.001	5
0–2 years old	0.4 (0.3–0.6)	69.4	<0.001	3
Overall mortality rate				
0–1 years old	1.5 (−1.1 to 4.1)	99.9	<0.001	2
0–2 years old	0.05 (0.03–0.07)	99.9	<0.001	4
Income levels^b^				
Incidence rate				
High‐income	15.0 (8.0–23.0)	100.0	<0.001	3
Low‐ and middle‐income^c^	7.0 (5.0–9.0)	99.5	<0.001	12
Hospitalization rate				
High‐income	1.7 (1.2–2.1)	100.0	<0.001	16
Low‐ and middle‐income^c^	1.2 (0.8–1.5)	99.3	<0.001	9
In‐hospital mortality rate				
High‐income	0.1 (0.1–0.2)	99.7	<0.001	5
Low‐ and middle‐income^c^	1.8 (0.5–3.1)	99.6	<0.001	4
Overall mortality rate				
High‐income	0.02 (0.01–0.02)	99.7	<0.001	2
Low‐ and middle‐income^c^	0.9 (0.6–1.2)	99.9	<0.001	3
Surveillance types				
Incidence rate				
Active surveillance	12.0 (8.0–17.0)	99.8	<0.001	8
Passive surveillance	3.0 (0.0–6.0)	99.7	<0.001	6
Data source				
In‐hospital mortality rate				
National based	0.1 (0.1–0.2)	99.8	<0.001	4
Hospital database	1.5 (0.6–2.3)	99.6	<0.001	5
Case definitions				
Incidence rate				
Extended ARI	10.0 (7.0–12.0)	99.8	<0.001	12
ILI	2.0 (1.0–3.0)	72.0	<0.001	3

Abbreviations: ARI, acute respiratory tract infection; CI, confidence interval; ILI, influenza‐like illness; RSV, respiratory syncytial virus.

^a^ 0–1 years old included children aged 1‐year‐old and younger, 0–2 years old included children aged 2‐years‐old and younger, 2–5 years old included 3‐, 4‐, and 5‐years‐old children.

b According to the 2021 World Bank classification for subgroup analysis.

^c^ Because limited studies focused on low‐income countries, we combined them with middle‐income countries as low‐ and middle‐income countries.

The pooled estimated RSV‐related disease incidence in 2–5‐year‐old children (3.0 per 100 children per year, 95% CI: 2.0–5.0) was significantly lower than that in 0–1‐year‐olds (9.0 per 100 children per year, 95% CI: 6.0–12.0) and 0–2‐year‐old children (9.0 per 100 children per year, 95% CI: 7.0–11.0), and the same trend was observed in RSV‐related hospitalization rate (2–5 years: 0.3 per 100 children per year, 95% CI: 0.2–0.4; 0–2 years: 1.2 per 100 children per year, 95% CI: 0.5–1.9; 0–1 years: 1.8 per 100 children per year, 95% CI: 1.3–2.3), in‐hospital mortality rate (0–2 years: 0.4 per 100 children per year, 95% CI: 0.3–0.6; 0–1 years: 1.1 per 100 children per year, 95% CI: 0.9–1.4), and overall mortality rate (0–2 years: 0.05 per 100 children per year, 95% CI: 0.03–0.07; 0–1 years: 1.5 per 100 children per year, 95% CI: −1.1 to 4.1).

According to the World Bank classification, the RSV‐related disease incidence in high‐income countries was higher than that in low‐ and middle‐income countries combined (15.0 per 100 children per year, 95% CI: 8.0–23.0 vs. 7.0 per 100 children per year, 95% CI: 5.0–9.0). The same trend was found in RSV‐related hospitalization rate (high‐income countries: 1.7 per 100 children per year, 95% CI: 1.2–2.1; low‐ and middle‐income countries combined: 1.2 per 100 children per year, 95% CI: 0.8–1.5). However, the opposite trend was observed in the RSV‐related in‐hospital mortality rate and overall mortality rate. The RSV‐related in‐hospital mortality rate in high‐income countries was lower than that in low‐ and middle‐income countries combined (0.1 per 100 children per year, 95% CI: 0.1–0.2 vs. 1.8 per 100 children per year, 95% CI: 0.5–3.1). The RSV‐related overall mortality in high‐income countries was lower than that in low‐ and middle‐income countries combined (0.02 per 100 children per year, 95% CI: 0.01–0.02 vs. 0.9 per 100 children per year, 95% CI: 0.6–1.2) (Table S8).

The pooled estimated RSV‐related disease incidence in active surveillance studies was significantly higher than that in passive surveillance studies (12.0 per 100 children per year, 95% CI: 8.0–17.0 vs. 3.0 per 100 children per year, 95% CI: 0–6.0). Moreover, the RSV‐related in‐hospital mortality rate was lower in studies that used nationwide databases (0.1 per 100 children per year, 95% CI: 0.1–0.2) and higher in studies conducted in single hospitals (1.5 per 100 children per year, 95% CI: 0.6–2.3). The estimated RSV‐related disease incidence was higher using the extended ARI‐based case definition than that estimated using the ILI‐based case definition (10.0 per 100 children per year, 95% CI: 7.0–12.0 vs. 2.0 per 100 children per year, 95% CI: 1.0–3.0).

### 3.5 Publication bias and sensitivity analysis

The results of the Egger's test did not show any significant publication bias (incidence: t = 2.3, *p* = 0.5; hospitalization rate: t = 1.6, *p* = 0.1, in‐hospital mortality rate: t = 4.3, *p* = 0.4; overall mortality rate: t = 2.6, *p* = 0.1). Details are shown in Figure S5. Sensitivity analyses of RSV‐related disease incidence, hospitalization rate, in‐hospital mortality rate, and overall mortality rate showed that, after applying the exclusion criteria, there was insignificant change in the estimated acceptance rate, suggesting the robustness of this analysis (Figure S6).

## 4 DISCUSSION

This meta‐analysis sought to comprehensively estimate the RSV‐related disease incidence, hospitalization rate, in‐hospital mortality rate, and overall mortality among children aged 5 years and younger. We aimed to provide basic data that could be used to develop a standardized surveillance system and make recommendations on vaccination of children aged 5 years and younger.

In our study, the pooled RSV‐related disease incidence in children aged 5 years and younger was considerably higher than that reported by Li et al. (9.0 per 100 children per year vs. 4.9 per 100 children per year).^7^ The previous article did not specifically report the burden of RSV‐ARI in primary care, whereas the studies included in our analysis to estimate the RSV‐related incidence were all from outpatients or active community surveillance, which could provide additional data. Furthermore, the higher incidence emphasizes the importance of strengthening surveillance in the community as well as in outpatient clinics.

The pooled RSV‐related hospitalization rate was 1.7 per 100 children per year in our study, which was considerably higher than that in the model study conducted by Li et al. in 2019 (0.5 per 100 children per year),^7^ and the pooled rate estimated by Stein et al. in 1989 to 2012 (0.4 per 100 children per year).^18^ On the other hand, the RSV‐related in‐hospital mortality rate (0.5 per 100 children per year) and overall mortality rate (0.05 per 100 children per year) among children aged 5 years and younger in our study were lower than the rates estimated by Li et al. in 2019 (0.7 per 100 children per year and 0.2 per 100 children per year, respectively).^7^ We assumed that an increasing hospitalization rate is not necessarily worrisome and it may reflect increased awareness of diagnosis and sufficient health care resources, which in turn can lead to a reduction in mortality.

To fully understand current studies on RSV‐related disease burden among young children, we included studies focused on the whole population and reported corresponding results of 0–5 years old children. Thus, we studied on children ≤5 years rather than <5 years as normally used. Meanwhile, the incidence of RSV‐related disease was likely low among 5 years old children, which was also confirmed in our subgroup analysis. Among children ≤5 years, RSV‐related disease incidence decreased with increasing age, and the incidence in the 0–1‐year‐old group was three times higher than that in the 2–5‐year‐old group (9.0 per 100 children per year vs. 3.0 per 100 children per year), which was consistent with the findings of Nair et al.^8^ Bardach et al. reported that the highest RSV‐positivity and mortality rates among the Latin‐American population were found in the 0–11‐months and 0–2‐years age groups, respectively.^19^ In Chinese children, the RSV‐positive rate was significantly higher in the 0–3‐year‐old group.^20^ In our study, the RSV‐related hospitalization rate was over six times higher in the 0–1‐year‐olds than that in the 2–5‐year‐olds (1.8 per 100 children per year vs. 0.3 per 100 children per year), the RSV‐related in‐hospital mortality rate was nearly three times higher in the 0–1‐year‐olds than that in 0–2‐year‐olds (1.1 per 100 children per year vs. 0.4 per 100 children per year), and the RSV‐related overall mortality rate of 0–1‐year‐olds was over twenty‐fold of that in the 0–2‐year‐olds (1.5 per 100 children per year vs. 0.05 per 100 children per year). Therefore, new‐born children and infants should be the primarily targeted population in the surveillance project.

The RSV‐related disease incidence in children aged 5 years and younger was significantly higher in high‐income countries (15.0 per 100 children per year) than that in low‐ and middle‐income countries combined (7.0 per 100 children per year). Nair and Li et al. estimated that over 95% of the global RSV‐related ALRI cases occurred in developing countries, whereas the positivity rate was higher in high‐income countries,^7^, ^8^ suggesting an underestimation of RSV‐related disease incidence in some low‐income countries. Our study found that higher income level was related with higher hospitalization rate, whereas the in‐hospital mortality and overall mortality rates decreased with increasing income level. Similarly, Li et al. found that, although RSV‐related hospitalization rates were slightly lower in low‐income countries than that in high‐income countries, the opposite was found for in‐hospital mortality rates (0.1 per 100 children per year vs. 0.8 per 100 children per year).^21^ Stein et al. also found that the hospitalization rate was slightly higher in developed countries than that in developing countries (1.9 per 100 children per year vs. 1.8 per 100 children per year), whereas the overall mortality rate was significantly higher in developing countries (2.1 per 100 children per year vs. 0.3 per 100 children per year).^18^ We presume that the main reason is that children in low‐ and middle‐income countries may tend to not visit the hospital unless they have severe disease. Li et al. observed that 97% of RSV‐attributable deaths occurred in low‐ and middle‐income countries, with the community mortality rate accounting for a higher proportion than the in‐hospital mortality rate,^7^ confirming that children in low‐ and middle‐income countries may have fewer opportunities to receive healthcare and that awareness of RSV infection diagnosis among clinicians was insufficient (Table S8). We also found that, in‐hospital mortality rates were considerably higher in studies with single hospital databases than those in multi‐center, long‐term, national studies (1.5 per 100 children per year vs. 0.1 per 100 children per year). In our study, all the data in national databases were collected in high‐income countries, further confirming that the economics could affect the disease burden of RSV in children aged 5 years and younger in multiple ways. A standard surveillance system will increase awareness of case ascertainment, and a unified data source is conducive to comparison among studies.

Methods of surveillance and case definitions were crucial points for a feasible surveillance system. In our study, the RSV‐related disease incidence was higher in active surveillance studies than that in passive surveillance studies (12.0 per 100 children per year vs. 3.0 per 100 children per year), consistent with the findings reported by Nair et al.^8^ This suggests that active surveillance could increase awareness of RSV infection among healthcare providers. From another perspective, it also means that passive surveillance may underestimate RSV‐related disease incidence. On the contrary, Kobayashi et al. found that active surveillance studies may underestimate hospitalization rates, compared with studies that retrospectively acquired hospitalization data based on case‐ascertainment through the use of International Classification of Diseases (ICD) codes.^22^ That may be due to restricting the inclusion criteria in active surveillance studies, which may have reduced the sensitivity of case ascertainment. For countries that have difficulty implementing active surveillance, using ICD codes may be a cost‐effective approach. In addition, our study found that the RSV‐related disease incidence was higher in studies that used extended ARI‐based case definition than that among those that used ILI‐based case definition. Considering that many children with RSV infection do not experience fever,^23^ the inclusion of temperature over 38°C in the ILI‐based case definition may lead to underestimation of the number of cases of RSV infection, especially in young children.^9^ The ILI‐based case definition is more specific, which can reduce false‐positive rates and save resources, whereas extended ARI symptoms are more sensitive and more suitable for estimating the comprehensive disease burden of RSV among children.^9^ Therefore, it is necessary to evaluate the balance between the sensitivity and the specificity of the surveillance method when considering how to construct a systematic RSV surveillance system and how to add RSV surveillance to the Global Influenza Surveillance and Response System.

This systematic review and meta‐analysis included many high‐quality original studies, among which 75% were ranked as high quality, and none were ranked as low quality. Additionally, we performed a specific and comprehensive subgroup analysis, which provides detailed information about the target high‐risk population and areas for RSV surveillance. The evaluation of various surveillance methods can provide scientific suggestions for constructing a standard and feasible surveillance system.

Our study also has some limitations. First, studies published in languages other than English and Chinese were excluded, which may lead to language bias. Second, the included studies had some obvious outliers and considerable heterogeneity owing to the differences in income level, age groups, surveillance types, case ascertainment standards, the diagnostic assays used to identify RSV, and the disparity in access to hospital care across studies. However, we did not exclude those studies considering that they met the inclusion criteria. We calculated the pooled rate and used a random‐effects model to synthesize the disease burden, which allows for some outliers and heterogeneity. The sensitivity analyses also confirmed the stability of the result. In addition, the aim of our study was to explore the influencing factors of heterogeneity and to eliminate the lack of comparability between studies in the future. We conducted a supplementary analysis of the studies that constituted the outliers in this section. Third, we only included studies relating to outpatients and community‐based surveillance to analyze RSV‐related incidence, which may have contributed to a higher incidence rate than that in other studies. However, this could provide a supplementary result for RSV‐related incidence in specific primary care settings. Finally, for the publication bias, studies did not conform well to the expected funnel shape, which may be due to the large heterogeneity and small number of the included studies. We further conducted a quantified Egger's test, and the results showed that there was no publication bias (all *p* > 0.05).

## 5 CONCLUSION

Our research provides an overall estimate of the RSV‐related disease burden in children aged 5 years and younger. More attention should be paid to RSV infection in children aged 2 years and younger, who are the target population of the surveillance project. Early detection and treatment can reduce disease severity and RSV‐related mortality. Awareness of RSV case ascertainment should be promoted among healthcare providers in low‐ and middle‐income countries. It is necessary to establish a feasible and standardized RSV surveillance system based on various considerations. Using the definition of extended ARI to determine patient inclusion will increase the sensitivity of case ascertainment. Active community surveillance is the top recommendation, whereas implementing a standard ICD code will be useful in retrospective passive surveillance and hospital‐based surveillance.

## AUTHORS CONTRIBUTIONS

*Concept and design*: Luzhao Feng and Weizhong Yang. *Acquisition of data*: Yuping Duan and Mingyue Jiang. *Analysis and interpretation of data*: Yuping Duan, Qiangru Huang, Mingyue Jiang, Mengmeng Jia, Luzhao Feng, and Weizhong Yang. *Drafting of the manuscript*: Yuping Duan and Mingyue Jiang. *Critical revision of the paper for important intellectual content*: Luzhao Feng and Weizhong Yang. *Statistical analysis*: Yuping Duan, Qiangru Huang, Mingyue Jiang, and Mengmeng Jia. *Obtaining funding*: Luzhao Feng and Weizhong Yang. *Administrative, technical, or logistic support*: Mingyue Jiang and Luzhao Feng. *Supervision*: Luzhao Feng and Weizhong Yang. All authors have read and agreed to the published version of the manuscript.

## CONFLICT OF INTEREST STATEMENT

The authors declare no conflict of interest. The funders had no role in the design of the study; in the collection, analyses, or interpretation of data; in the writing of the manuscript; or in the decision to publish the results.

### PEER REVIEW

The peer review history for this article is available at https://www.webofscience.com/api/gateway/wos/peer-review/10.1111/irv.13145.

## Supporting information


**Table S1.** Search strategy.
**Table S2.** Related definitions.
**Table S3.** Summary of studies that contributed to RSV‐related incidence rate.
**Table S4.** Summary of studies that contributed to RSV‐related hospitalization rate.
**Table S5.** Summary of studies that contributed to RSV‐related in‐hospital mortality rate.
**Table S6.** Summary of studies that contributed to RSV‐related overall mortality.
**Figure S1.** Subgroup analysis of RSV‐related incidence among children ≤5 years old.
**Figure S2.** Subgroup analysis of RSV‐related hospitalization rate among children ≤5 years old.
**Figure S3.** Subgroup analysis of RSV‐related in‐hospital mortality rate among children ≤5 years old.
**Figure S4.** Subgroup analysis of RSV‐related overall mortality rate among children ≤5 years old.
**Figure S5.** Publication bias analyzed by funnel plot.
**Figure S6.** Sensitivity analysis.
**Table S7.** Quality scoring criteria for observational study studies.
**Table S8.** Comparation of RSV‐related disease burden in different studies.
Outliers' analysis.

## Data Availability

The data that support the findings of this study are available from the corresponding author, Luzhao Feng, upon reasonable request.
