# Supplementary material for: Incidence, hospitalization, and mortality in children aged 5 years and younger with respiratory syncytial virus‐related diseases: A systematic review and meta‐analysis
Source: Influenza Other Respir Viruses. 2023 May 22;17(5):e13145. doi: 10.1111/irv.13145 (PMC10201211; doi:10.1111/irv.13145)
Supplement: Supplementary file 1 — Table S1. Search strategy. Table S2. Related definitions. Table S3. Summary of studies that contributed to RSV‐related incidence rate. Table S4. Summary of studies that contributed to RSV‐related hospitalization rate. Table S5. Summary of studies that contributed to RSV‐related in‐hospital mortality rate. Table S6. Summary of studies that contributed to RSV‐related overall mortality. Figure S1. Subgroup analysis of RSV‐related incidence among children ≤5 years old. Figure S2. Subgroup analysis of RSV‐related hospitalization rate among children ≤5 years old. Figure S3. Subgroup analysis of RSV‐related in‐hospital mortality rate among children ≤5 years old. Figure S4. Subgroup analysis of RSV‐related overall mortality rate among children ≤5 years old. Figure S5. Publication bias analyzed by funnel plot. Figure S6. Sensitivity analysis. Table S7. Quality scoring criteria for observational study studies. Table S8. Comparation of RSV‐related disease burden in different studies. Outliers' analysis. [file IRV-17-e13145-s001.docx]

**Supplementary appendix:**

**Supplementary table 1. Search strategy.**

| CNKI |
| --- |
| (SU=Respiratory Syncytial Virus OR SU=Respiratory Syncytial Virus Infections OR SU=Human Respiratory Syncytial Virus OR SU=Human orthopulmonary virus OR SU=RSV OR SU=HRSV) AND (SU=Child OR SU=Infant OR SU=Pediatrics OR SU=Toddler OR SU=Baby OR SU=Newborn OR SU=Neonat OR SU=kindergarten OR SU=Preschool OR SU=preschool child OR SU=schoolchild OR SU=school age OR SU=Neonatology department OR SU=Pediatric department OR SU=nurser) AND (SU=Incidence rate OR SU=Incidence OR SU=Prevalence rate OR SU=Prevalence OR SU=Infection rate OR SU=Infection OR SU=Positive rate of serum antibody OR SU=Positive serum antibody OR SU=Outpatient rate OR SU=Visiting rate OR SU=Admission rate OR SU=Hospitalization rate OR SU=Case fatality rate OR SU=Mortality OR SU=Death) |
| 1852 records |
| CBM |
| #1"Respiratory Syncytial Virus"[Unweighted: extended] OR "Respiratory Syncytial Virus, Human"[Unweighted: extended]  4058  #2"Respiratory Syncytial Virus"[Common words: Intelligence] OR "Respiratory Syncytial Virus Infections"[Common words: Intelligence] OR "Human Respiratory Syncytial Virus Infections"[Common words: Intelligence] OR "Human orthopulmonary virus"[Common words: Intelligence] OR "RSV"[Common words: Intelligence] OR "HRSV"[Common words: Intelligence]  [27326](javascript:historyLink(')  #3#1 OR #2  27326  #4((("Child"[Unweighted: extended]) OR "Infant, Newborn "[Unweighted: extended]) OR "Toddler"[Unweighted: extended]) OR "Child, Preschool"[Unweighted: extended]  197302  #5"Child"[Common words: Intelligence] OR "Infant"[Common words: Intelligence] OR "Pediatric"[Common words: Intelligence] OR "Toddler"[Common words: Intelligence] OR "Baby"[Common words: Intelligence] OR "child patient"[Common words: Intelligence] OR "Newborn "[Common words: Intelligence] OR "Pediatric department"[Common words: Intelligence] OR "Neonatology department"[Common words: Intelligence]  2918169  #6#4 OR #5  2918169  #7(("Incidence"[Unweighted: extended]) OR "Prevalence"[Unweighted: extended]) OR "Infection"[Unweighted: extended]  3358879  #8"Incidence rate"[Common words: Intelligence] OR "Incidence"[Common words: Intelligence] OR "Prevalence rate"[Common words: Intelligence] OR "Prevalence"[Common words: Intelligence] OR "Infection rate"[Common words: Intelligence] OR "Infection"[Common words: Intelligence] OR "Positive rate of serum antibody"[Common words: Intelligence] OR "Positive serum antibody"[Common words: Intelligence]  [6368811](javascript:historyLink(')  #9#7 OR #8  6368811  #10"Morbidity"[Unweighted: extended] OR "Death"[Unweighted: extended]  [672374](javascript:historyLink(')  #11"Outpatients rate"[Common words: Intelligence] OR "Visiting rate"[Common words: Intelligence] OR "Admission rate"[Common words: Intelligence] OR "Hospitalization rate"[Common words: Intelligence] OR "Case fatality rate"[Common words: Intelligence] OR "Morbidity"[Common words: Intelligence] OR "Death"[Common words: Intelligence]  3897196  #12#10 OR #11  3897196  #13#9 OR #12  9240463  #14#3 AND #6 AND #13 AND 2010-2022[date]  6857 |
| 2690 records |
| Vip |
| ((((M=Respiratory Syncytial Virus OR M=Respiratory Syncytial Virus Infections) OR M=Respiratory Syncytial Virus，Human) AND (((((((((((((((M=Child OR M=Infant) OR M=Pediatrics) OR M=Neonat) OR M=Baby) OR M=Child patient) OR M=Newborn) OR M=Pediatric department) OR M=Neonatology department) OR M=Infancy) OR M=Toddler) OR M=Preschool) OR M=kindergarten) OR M=nurser) OR M=preschool child) OR M=5 years old)) AND ((((((((((((((M=Incidence rate OR M=Incidence) OR M= Incidence rate) OR M=Prevalence ) OR M=Infection rate) OR M=Infection ) OR M=Positive rate of serum antibody) OR M=Positive serum antibody) OR M=Outpatient rate) OR M=Visiting rate) OR M=Hospitalization rate) OR M=Hospitalization) OR M=Case fatality rate) OR M=Mortality) OR M=Death)) |
| 455 records |
| Wanfang |
| theme:("Respiratory Syncytial Virus" or "Respiratory Syncytial Virus Infections " or "Respiratory Syncytial Virus，Human ") and theme:("Child" or "Infant" or "Pediatrics" or "Neonat" or "Baby" or "Child patient" or "Newborn" or "Pediatric department" or "Neonatology department" or "Infancy" or "Toddler" or "Preschool" or "kindergarten" or "preschool child") and theme:("Incidence rate" or "Incidence" or "Prevalence rate" or "Prevalence" or "Infection rate" or "Infection" or "Positive rate of serum antibody" or "Positive serum antibody" or "Outpatient rate" or "Visiting rate" or "Hospitalization rate" or "Hospitalization" or "Case fatality rate" or "Mortality" or "Death") |
| 2965 records |
| Pubmed |
| 1.(("Respiratory Syncytial Virus Infections"[Mesh]) OR "Respiratory Syncytial Viruses"[Mesh]) OR "Respiratory Syncytial Virus, Human"[Mesh] Filters: from 2010/1/1 - 2022/6/2  5865  2.((((((respiratory Syncytial Virus Infection*[Title/Abstract]) OR (human respiratory syncytial virus [Title/Abstract])) OR (RSV[Title/Abstract])) OR (respiratory syncytial virus*[Title/Abstract])) OR (HRSV[Title/Abstract])) OR (RS virus[Title/Abstract])) OR (RS-v[Title/Abstract]) Filters: from 2010/1/1 - 2022/6/2  10657  3.1 OR 2  11043  4.(((((("Child, Preschool"[Mesh]) OR "Child"[Mesh]) OR "Infant"[Mesh]) OR "Pediatrics"[Mesh]) OR "Schools, Nursery"[Mesh]) OR "Nurseries, Infant"[Mesh]) OR "Nurseries, Hospital"[Mesh] Filters: from 2010/1/1 - 2022/6/2  940886   1. (((((((((((((Child*[Title/Abstract]) OR (infant*[Title/Abstract])) OR (pediatric*[Title/Abstract])) OR (Toddler*[Title/Abstract])) OR (Kindergarten*[Title/Abstract])) OR (Preschool*[Title/Abstract])) OR (Baby[Title/Abstract])) OR (babies[Title/Abstract])) OR (Newborn*[Title/Abstract])) OR (Neonat*[Title/Abstract])) OR (pre-school*[Title/Abstract])) OR (nurser*[Title/Abstract])) OR (schoolchild*[Title/Abstract])) OR (school age*[Title/Abstract]) Filters: from 2010/1/1 - 2022/6/2   1085486  6.4 OR 5  1356545  7.(("Incidence"[Mesh]) OR "Morbidity"[Mesh]) OR "Prevalence"[Mesh] Filters: from 2010/1/1 - 2022/6/2  353971  8.((((((((((incidence[Title/Abstract]) OR (Morbidity[Title/Abstract])) OR (attack rate[Title/Abstract])) OR (incidence rate[Title/Abstract])) OR (prevalence rate[Title/Abstract])) OR (prevalence[Title/Abstract])) OR (sickness rate[Title/Abstract])) OR ("infection rate"[Title/Abstract])) OR (antibody positive[Title/Abstract])) OR (Serum antibody positive rate[Title/Abstract])) OR (occurrence[Title/Abstract]) Filters: from 2010/1/1 - 2022/6/2  1284003  9.7 OR 8 Filters: from 2010/1/1 - 2022/6/2  1364722   1. ((("Inpatients"[Mesh]) OR "Hospitalization"[Mesh]) OR "Mortality"[Mesh]) Filters: from 2010/1/1 - 2022/6/2   350612  11.(((((((((outpatient rate[Title/Abstract]) OR (visiting rate[Title/Abstract])) OR (admission rate[Title/Abstract])) OR (hospitalization rate[Title/Abstract])) OR (Inpatient*[Title/Abstract])) OR (fatality rate[Title/Abstract])) OR (case fatality rate[Title/Abstract])) OR (death rate[Title/Abstract])) OR (mortality[Title/Abstract])) OR (death[Title/Abstract]) Filters: from 2010/1/1 - 2022/6/2  1023030  12.10 OR 11 Filters: from 2010/1/1 - 2022/6/2  1205027  13. 9 OR 12 Filters: from 2010/1/1 - 2022/6/2  2214179  14.3 AND 6 AND 13 Filters: from 2010/1/1 - 2022/6/2 Filters: from 2010/1/1 - 2022/6/2  2889  15 "Humans"[MeSH Terms] Filters: from 2010/1/1 - 2022/6/2  8206357  16."human"[Title/Abstract] OR "humans"[Title/Abstract] Filters: from 2010/1/1 - 2022/6/2  1483880  17.15 OR 16 Filters: from 2010/1/1 - 2022/6/2  8613457  18.14 AND 17 Filters: from 2010/1/1 - 2022/6/2 |
| 2640 records |
| Embase |
| #1 'respiratory syncytial virus infection'/exp OR 'pneumovirus'/exp OR 'human respiratory syncytial virus'/exp  25918  #2 'respiratory syncytial virus infection*':ab,ti OR 'human respiratory syncytial virus':ab,ti OR 'rsv':ab,ti OR 'respiratory syncytial virus*':ab,ti OR hrsv:ab,ti OR 'rs virus':ab,ti  26150  #3 #1 OR #2  33451  #4 'preschool child'/exp OR 'child'/exp OR 'infant'/exp OR 'pediatrics'/exp OR 'nursery school'/exp OR 'nursery'/exp  3306656  #5 child*:ab,ti OR infant*:ab,ti OR pediatric*:ab,ti OR toddler*:ab,ti OR kindergarten*:ab,ti OR preschool*:ab,ti OR baby:ab,ti OR babies:ab,ti OR newborn*:ab,ti OR neonat*:ab,ti OR 'pre school*':ab,ti OR nurser*:ab,ti OR schoolchild*:ab,ti OR 'school age*':ab,ti  3067831  #6 #4 OR #5  4220400  #7 'incidence'/exp OR 'morbidity'/exp OR 'prevalence'/exp  1824331  #8 incidence:ab,ti OR morbidity:ab,ti OR 'attack rate':ab,ti OR 'incidence rate':ab,ti OR 'prevalence rate':ab,ti OR prevalence:ab,ti OR 'sickness rate':ab,ti OR 'incidence rate ratio':ab,ti OR 'infection rate':ab,ti OR 'antibody positive':ab,ti OR 'serum antibody positive rate':ab,ti OR occurrence:ab,ti  3184839  #9 #7 OR #8  3622568  #10 'hospital patient'/exp OR 'hospitalization'/exp OR 'mortality'/exp  1837477  #11 'outpatient rate':ab,ti OR 'visiting rate':ab,ti OR 'admission rate':ab,ti OR 'hospitalization rate':ab,ti OR inpatient*:ab,ti OR 'severe rate':ab,ti OR 'fatality rate':ab,ti OR 'case fatality rate':ab,ti OR 'death rate':ab,ti OR mortality:ab,ti OR death:ab,ti  2513460  #12 #10 OR #11  3232447  #13 #9 OR #12  5860715  #14 #3 AND #6 AND #13  7670  #15 #14 AND (2010:py OR 2011:py OR 2012:py OR 2013:py OR 2014:py OR 2015:py OR 2016:py OR 2017:py OR 2018:py OR 2019:py OR 2020:py OR 2021:py OR 2022:py)  5544  #16 'human'/exp  25415287  #17 human*:ab,ti  3802878  #18 #16 OR #17  26272545  #19 #15 AND #18  5350  #20 #19 AND [embase]/lim NOT ([embase]/lim AND [medline]/lim) |
| 2148 records |
| The Cochrane library |
| 1.MeSH descriptor: [Respiratory Syncytial Virus Infections] explode all trees  243  2.MeSH descriptor: [Respiratory Syncytial Virus, Human] explode all trees  81  3.MeSH descriptor: [Respiratory Syncytial Viruses] explode all trees  193  4.(respiratory Syncytial Virus Infection* OR human respiratory syncytial virus OR RSV OR respiratory syncytial virus* OR HRSV):ti,ab,kw AND (enteral nutrition):ti,ab,kw  3  5.#1 OR #2 OR #3 OR #4  365  6.MeSH descriptor: [Child, Preschool] explode all trees  31618  7.MeSH descriptor: [Child] explode all trees  62178  8.MeSH descriptor: [Infant] explode all trees  35271  9.MeSH descriptor: [Pediatrics] explode all trees  729  10.MeSH descriptor: [Nurseries, Infant] explode all trees  12  11.MeSH descriptor: [Nurseries, Hospital] explode all trees  35  12.MeSH descriptor: [Nurseries, Infant] explode all trees  12  13.(Child* OR infant* OR pediatric* OR Toddler* OR Kindergarten* OR Preschool* OR Baby OR babies OR Newborn* OR Neonat* OR pre-school* OR nurser* OR schoolchild* OR school age*):ti,ab,kw  239952  14.#6 OR #7 OR #8 OR #9 OR #10 OR #11 OR #12 OR #13  239958  15.#5 AND #14  265  16.(infections OR infection rate OR prevalence rate OR Incidence OR Morbidity OR attack rate OR incidence rate OR prevalence rate OR Prevalence OR sickness rate OR antibody positive OR Serum antibody positive rate OR occurrence OR outpatient rate OR visiting rate OR admission rate OR hospitalization rate OR Inpatient* OR fatality rate OR case fatality rate OR death rate OR mortality ):ti,ab,kw  383704  17.MeSH descriptor: [Incidence] OR [Morbidity] OR[Prevalence] explode all trees  0  18.#16 OR #17  383704  19.#15 AND #18 with Publication Year from 2010 to 2022, |
| 156 records |

**Supplementary table 2. Related definitions.**

|  | Definitions |
| --- | --- |
| Active surveillance | Community and/or sentinel hospitals regularly report cases to superior department according to uniform standards ^1^. Based on the practical implementation of studies included, our study further defined it as the detection of RSV infected children by health workers through regular house-to-house follow-up ^2^. |
| Passive surveillance | Researchers do not actively detect and track cases, data are mostly taken retrospectively from national surveillance databases, hospital databases and laboratory confirmed cases ^1^. |
| Influenza-like illness (ILI) | It refers to cases with fever (body temperature ≥38℃) accompanied by cough or sore throat ^3^. |
| Extensional Acute respiratory tract infection (ARI) | Acute onset of infectious symptoms with at least one respiratory symptom of cough, sore throat, difficult or laboured breathing or coryza ^1^. Including any symptoms associated with respiratory tract infections such as upper respiratory tract infections (URTI), lower respiratory tract infections (LRTI) and severe acute respiratory tract infections (SARI) etc. |
| World Bank income regions | The World Bank's income classifications split countries into one of four categories determined by the country's gross national income (GNI) per capita in US$. The GNI thresholds between income groups has changed through time based on World Bank definitions^4^. We combined the lower-middle-income and the upper-middle-income countries as middle-income countries, and the countries included in our study are further divided into the following three categories: high-income countries (such as Norway, Finland, the United Kingdom, the United States, Germany, Japan, Spain, Sweden, France, New Zealand, Italy, Mexico etc.), middle-income countries (such as Nepal, Kenya, Ecuador, India, Egypt, Indonesia, Bangladesh, Jordan, Argentina, Thailand, Bolivia, China etc.), and low-income countries (such as Gambia, Madagascar, Zambia etc.) |

**Supplementary table 3. Summary of studies that contributed to RSV-related incidence rate.**

| **No** | **The first author** | **Study Period** | **Country** | [**Race**](javascript:;) | [**Study type**](javascript:;) | **Data sources** | **Symptoms** | **Specimen** | **Diagnostic test** | **Sample size** | **Ages** | **Gender (male/female)** | **QA score** |
| --- | --- | --- | --- | --- | --- | --- | --- | --- | --- | --- | --- | --- | --- |
| 1 | L. B. Havda^6^ | 2015-2018 | Norway | n/a | Passive surveillance | National based | ARI | NPS | PCR | 2,590 | 0-5Y | 1284/1008 | 10 |
| 2 | L. Toivonen^7^ | 2008-2010 | Finland | n/a | Active surveillance | Birth-cohort | ARI | NS | PCR | 4,728 | 0-2Y | 488/435 | 8 |
| 3 | J. Boonyaratanakornkit^8^ | 2011-2014 | Nepal | n/a | Active surveillance | Birth-cohort | ARI | NS | PCR | 3,528 | 0-6M | 1861/1667 | 8 |
| 4 | R. F. Breiman^9^ | 2007-2011 | Kenya | n/a | Active surveillance | National based | SARI | NPS and OPS | PCR | 2,592 | 0-5Y | 1369/1223 | 8 |
| 5 | H. Y. Chu^10^ | 2011-2014 | Nepal | n/a | Active surveillance | National based | ARI | NS | PCR | 3,509 | 0-6M | 1851/1658 | 10 |
| 6 | E. Azziz‐Baumgartner^11^ | 2011-2014 | Ecuador | Afro-Ecuadorian: 607；  Mestizo: 1760；  Indigenous: 9 | Active surveillance | Birth-cohort | ARI | NPS | PCR | 1,289 | 12-95M | 1216/1160 | 8 |
| 7 | E. A. Okiro^12^ | 2002-2004 | Kenya | n/a | Active surveillance | National based | ARI | NS | IF | 2,143 | 0-5Y | 1069/1094 | 10 |
| 8 | Y. Kobayashi^13^ | 2017-2018 | Japan | n/a | Retrospective observational cohort | National based | Medical requirement  RSV | n/a | n/a | 18,220 | 0-2Y | 10001/8219 | 7 |
| 9 | A. Satav^14^ | 2016-2020 | India | n/a | Active surveillance | National based | SARI | NPS | n/a | 5,997 | 0-2Y | 3041/2956 | 7 |
| 10 | E. Rowlinson^15^ | 2009-2012 | Egypt | n/a | Passive surveillance | National based | ILI or ARI | NPS and OPS | PCR | 1,678 | 1M-4Y | 985/693 | 8 |
| 11 | G. O. Emukule^16^ | 2009-2012 | Kenya | n/a | Passive surveillance | National based | ILI or SARI | NPS and OPS | PCR | 3,322 | 0-4Y | 1664/1658 | 9 |
| 12 | E. A. Simões^17^ | 1999-2001 | Indonesia | n/a | Active surveillance | Birth-cohort | ARI | NLF | PCR | 2,014 | 0-5Y | 1021/993 | 8 |
| 13 | L. Lu^18^ | 2015 | Gambia | n/a | Active surveillance | National based | ARI | NPS and OPS | PCR | 2,385 | 2M-2Y | 300/236 | 8 |
| 14 | F. P. Havers^19^ | 2004-2008 | Bangladesh | n/a | Active surveillance | National based | ARI | NLF | PCR | 17,644 | 0-5Y | n/a | 9 |
| 15 | G. M. Bigogo^20^ | 2007-2011 | Kenya | n/a | Passive and active surveillance | National based | SARI | NPS or OPS | PCR | 27,269 | 0-5Y | 400/421 | 9 |

RSV = respiratory syncytial virus; ARI = acute respiratory infection; ILI = influenza-like illness; SARI = severe acute respiratory tract infection; LRTI = lower respiratory tract infection; NPS = nasopharyngeal swab; NS = nose swab; OPS = oropharyngeal swab; NLF = nasal lavage fluid; PCR = polymerase chain reaction; IF = immunofluorescence; QA = quality assessment; n/a = not analyzed; Y = Year; M = Month.

**Supplementary table 4. Summary of studies that contributed to RSV-related hospitalization rate.**

| **No** | **The first author** | **Study Period** | **Country** | [**Race**](javascript:;) | [**Study type**](javascript:;) | **Data sources** | **Symptoms** | **Specimen** | **Diagnostic test** | **Sample size** | **Ages** | **Gender (male/female)** | **QA score** |
| --- | --- | --- | --- | --- | --- | --- | --- | --- | --- | --- | --- | --- | --- |
| 1 | L. B. Havdal^6^ | 2015-2018 | Norway | n/a | Passive surveillance | National based | ARI | NPS | PCR | 2,590 | 0-5Y | 1284/1008 | 10 |
| 2 | R. Gil-Prieto^21^ | 1997-2011 | Spain | n/a | Passive surveillance | National based | Bronchitis | n/a | n/a | 30,417,106 | 0-5Y | n/a | 7 |
| 3 | C. S. Arriola^22^ | 2014-2015 | America | Hispanic: 360；  White non-Hispanic: 494；  Black non-Hispanic: 375；  Other: 168；  Missing data: 157 | Passive surveillance | National based | RSV confirmed cases | n/a | PCR, rapid antigen  Test, fluorescent antibody, or viral culture | 1,554 | 0-2Y | 895/659 | 10 |
| 4 | C. Muñoz-Quiles  ^23^ | 2009-2012 | Spain | n/a | Passive surveillance | National based | Bronchitis | n/a | n/a | 41,479 | 0-2Y | 24361/17118 | 9 |
| 5 | R. Thwaites^24^ | 2000-2011 | America | n/a | Passive surveillance | National based | SARI | n/a | n/a | 13,362 | 0-2Y | 7554/11808 | 8 |
| 6 | C. Svensson^25^ | 2004-2011 | Sweden | n/a | Passive surveillance | National based | ARI | NPS | rapid antigen detection | 52,781 | 0-4Y | n/a | 8 |
| 7 | N. Khuri-Bulos^26^ | 2010-2013 | Jordan | n/a | Passive surveillance | Hospital database | ARI | NPS and OPS | PCR | 3,168 | 0-2Y | 1912/1256 | 8 |
| 8 | H. Chi^27^ | 2004-2007 | Taiwan | n/a | Passive surveillance | Hospital database | ARI | n/a | n/a | 11,081 | 0-5Y | 276/194 | 7 |
| 9 | D. N. Marcone^28^ | 2008-2010 | Argentina | n/a | Passive surveillance | Hospital database | ARI | NPS | IF | 4,739 | 0-5Y | 169/189 | 8 |
| 10 | C. Demont^29^ | 2010-2018 | France | n/a | Passive surveillance | National based | ARI | n/a | n/a | 23,835,288 | 0-5Y | n/a | 8 |
| 11 | R. Kramer^30^ | 2012-2016 | France | n/a | Passive surveillance | Hospital database | ARI | n/a | n/a | 21,930 | 0-1Y | n/a | 7 |
| 12 | A. M. Helfrich^31^ | 2005-2011 | Argentina | n/a | Passive surveillance | National based | ARI | n/a | n/a | 599,535 | 0-1Y | n/a | 8 |
| 13 | L. M. Yoshida^32^ | 2007-2010 | Vietnam | n/a | Passive surveillance | Hospital database | ARI | NPS | PCR | 1,992 | 0-5Y | 1211/781 | 10 |
| 14 | N. Halasa^33^ | 2010-2013 | Jordan | n/a | Passive surveillance | Hospital database | ARI | NS or OPS | PCR | 3,168 | 0-2Y | 1912/1256 | 8 |
| 15 | G. O. Emukule^16^ | 2009-2012 | Kenya | n/a | Passive surveillance | National based | SARI | NPS and OPS | PCR | 3,322 | 0-4Y | 1664/1658 | 9 |
| 16 | M. V. Bennett^34^ | 1997-2011 | America | n/a | Passive surveillance | Hospital database | ARI | n/a | n/a | 7,177,171 | 0-1Y | 3670350/3506807 | 9 |
| 17 | N. Prasad^35^ | 2012-2015 | New Zealand | Māori: 1617；  Pacific: 2348；  Asian: 496；  European/other: 848 | Passive surveillance | National based | ARI | NPS | PCR | 5,309 | 0-5Y | n/a | 8 |
| 18 | P. Kuhdari^36^ | 2001-2014 | Italy | n/a | Passive surveillance | National based | ARI | n/a | n/a | 361,190 | 0-4Y | n/a | 6 |
| 19 | S. Naorat^37^ | 2008-2011 | Thailand | n/a | Passive surveillance | Hospital database | ARI | NPS | PCR | 54,311 | 全人群 | 30077/24234 | 9 |
| 20 | L. Cattoir ^38^ | 2012-2017 | Bolivia | n/a | Passive surveillance | National based | SARI | n/a | n/a | 1,483 | 0-4Y | n/a | 7 |
| 21 | J. H. Rabarisonl^39^ | 2011-2016 | Madagascar | n/a | Passive surveillance | Hospital database | SARI | NPS and OPS | PCR | 6,170 | 0-5Y | n/a | 8 |
| 22 | M. B. Jalink^40^ | 1998-2009 | Canada | n/a | Passive surveillance | National based | ARI | n/a | n/a | 3,916 | 0-5Y | 2098/1818 | 9 |
| 23 | H. C. Moore^41^ | 2000-2012 | Australian | n/a | Passive surveillance | National based | RSV confirmed cases | Respiratory specimens | IF, PCR or viral culture | 64,382 | 0-16Y | n/a | 8 |
| 24 | Z. Straňák^42^ | 2013-2014 | 23 countries | White: 1846；  Black: 42；  Asian: 161；  Mixed/Other: 337；  Missing: 4 | Passive surveillance | National based | ARI | n/a | n/a | 2,390 | 0-6M | 1255/1135 | 9 |
| 25 | E. Rowlinson^15^ | 2009-2012 | Egypt | n/a | Passive surveillance | National based | ARI | NPS and OPS | PCR | 1,678 | 1M-4Y | 985/693 | 8 |

RSV = respiratory syncytial virus; ARI = acute respiratory infection; ILI = influenza-like illness; SARI = severe acute respiratory tract infection; LRTI = lower respiratory tract infection; NPS = nasopharyngeal swab; NS = nose swab; OPS = oropharyngeal swab; NLF = nasal lavage fluid; PCR = polymerase chain reaction; IF = immunofluorescence; QA = quality assessment; n/a = not analyzed; Y = Year; M = Month.

**Supplementary table 5****. Summary of studies that contributed to RSV-related in-hospital mortality rate.**

| **No** | **The first author** | **Study Period** | **Country** | [**Race**](javascript:;) | [**Study type**](javascript:;) | **Data sources** | **Symptoms** | **Specimen** | **Diagnostic test** | **Sample size** | **Ages** | **Gender (male/female)** | **QA score** |
| --- | --- | --- | --- | --- | --- | --- | --- | --- | --- | --- | --- | --- | --- |
| 1 | R. Gil-Prieto^21^ | 1997-2011 | Spain | n/a | Passive surveillance | National based | bronchitis | n/a | n/a | 30,417,106 | 0-5Y | n/a | 7 |
| 2 | C. L. Byington^43^ | 2000-2011 | America | n/a | Passive surveillance | National based | ARI | n/a | n/a | 872,658 | 0-2Y | n/a | 8 |
| 3 | C. S. Arriola^22^ | 2014-2015 | America | Hispanic: 360；  White non-Hispanic: 494；  Black non-Hispanic: 375；  Other: 168；  Missing data: 157 | Passive surveillance | National based | RSV confirmed cases | n/a | PCR, rapid antigen detection, fluorescent antibody, or viral culture | 1,554 | 0-2Y | 895/659 | 10 |
| 4 | MI Pei-ming ^44^ | 2018-2019 | China | n/a | Passive surveillance | Hospital database | Pneumonia | OPS | Double amplification | 5,946 | 0-2Y | n/a | 7 |
| 5 | H. Chi^27^ | 2004-2007 | Taiwan | n/a | Passive surveillance | Hospital database | ARI | n/a | n/a | 11,081 | 0-5Y | 276/194 | 7 |
| 6 | ZHANG Xiao-bo ^45^ | 2012-2013 | China | n/a | Passive surveillance | Hospital database | ARI | Airway suction fluid | IF | 1,726 | 0-1Y | 1150/575 | 7 |
| 7 | C. Demont^29^ | 2010-2018 | France | n/a | Passive surveillance | National based | ARI | n/a | n/a | 23,835,288 | 0-5Y | n/a | 8 |
| 8 | N. Halasa^33^ | 2010-2013 | Jordan | n/a | Passive surveillance | Hospital database | ARI | NS or OPS | PCR | 3,168 | 0-2Y | 1912/1256 | 8 |
| 9 | S. Vizcarra-Ugalde^46^ | 2003-2014 | Mexico | n/a | Passive surveillance | Hospital database | RSV confirmed cases | NPS | IF or PCR | 1,153 | 0-5Y | n/a | 7 |

RSV = respiratory syncytial virus; ARI = acute respiratory infection; ILI = influenza-like illness; SARI = severe acute respiratory tract infection; LRTI = lower respiratory tract infection; NPS = nasopharyngeal swab; NS = nose swab; OPS = oropharyngeal swab; NLF = nasal lavage fluid; PCR = polymerase chain reaction; IF = immunofluorescence; QA = quality assessment; n/a = not analyzed; Y = Year; M = Month.

**Supplementary table 6****. Summary of studies that contributed to RSV-related overall mortality.**

| **No** | **The first author** | **Study Period** | **Country** | [**Race**](javascript:;) | [**Study type**](javascript:;) | **Data sources** | **Symptoms** | **Specimen** | **Diagnostic test** | **Sample size** | **Ages** | **Gender (male/female)** | **QA score** |
| --- | --- | --- | --- | --- | --- | --- | --- | --- | --- | --- | --- | --- | --- |
| 1 | R. Gil-Prieto^21^ | 1997-2011 | Spain | n/a | Passive surveillance | National based | Bronchitis | n/a | n/a | 30,417,106 | 0-5Y | n/a | 7 |
| 2 | C. L. Byington^43^ | 2000-2011 | America | n/a | Passive surveillance | National based | ARI | n/a | n/a | 872,658 | 0-2Y | n/a | 8 |
| 3 | E. A. F. Simões^47^ | 2016-2020 | India | n/a | Active surveillance | National based | LRTI | NPS | PCR | 23,763 | 0-2Y | 7122/16641 | 9 |
| 4 | C. J. Gill^48^ | 2017-2020 | Zambia | n/a | Passive surveillance | National based | RSV confirmed cases | NPS | PCR | 2,286 | 4D-6M | 1034/1069/183 | 10 |
| 5 | F. M. Ferolla^49^ | 2011 | Argentina | n/a | Passive surveillance | Hospital database | SARI | NS | PCR | 1,293 | 0-2Y | 678/615 | 8 |

RSV = respiratory syncytial virus; ARI = acute respiratory infection; ILI = influenza-like illness; SARI = severe acute respiratory tract infection; LRTI = lower respiratory tract infection; NPS = nasopharyngeal swab; NS = nose swab; OPS = oropharyngeal swab; NLF = nasal lavage fluid; PCR = polymerase chain reaction; IF = immunofluorescence; QA = quality assessment; n/a = not analyzed; Y = Year; M = Month.

| 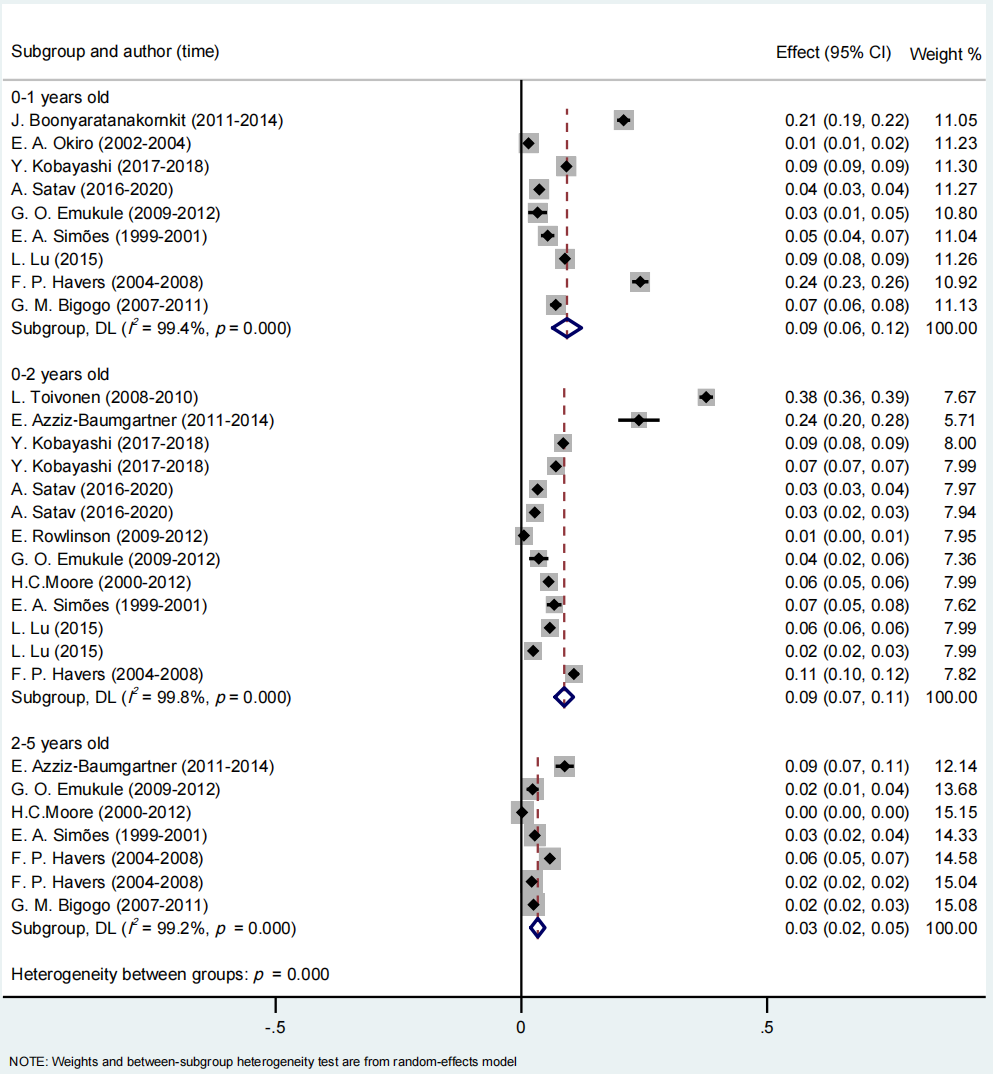 |
| --- |
| **Ages** |
| 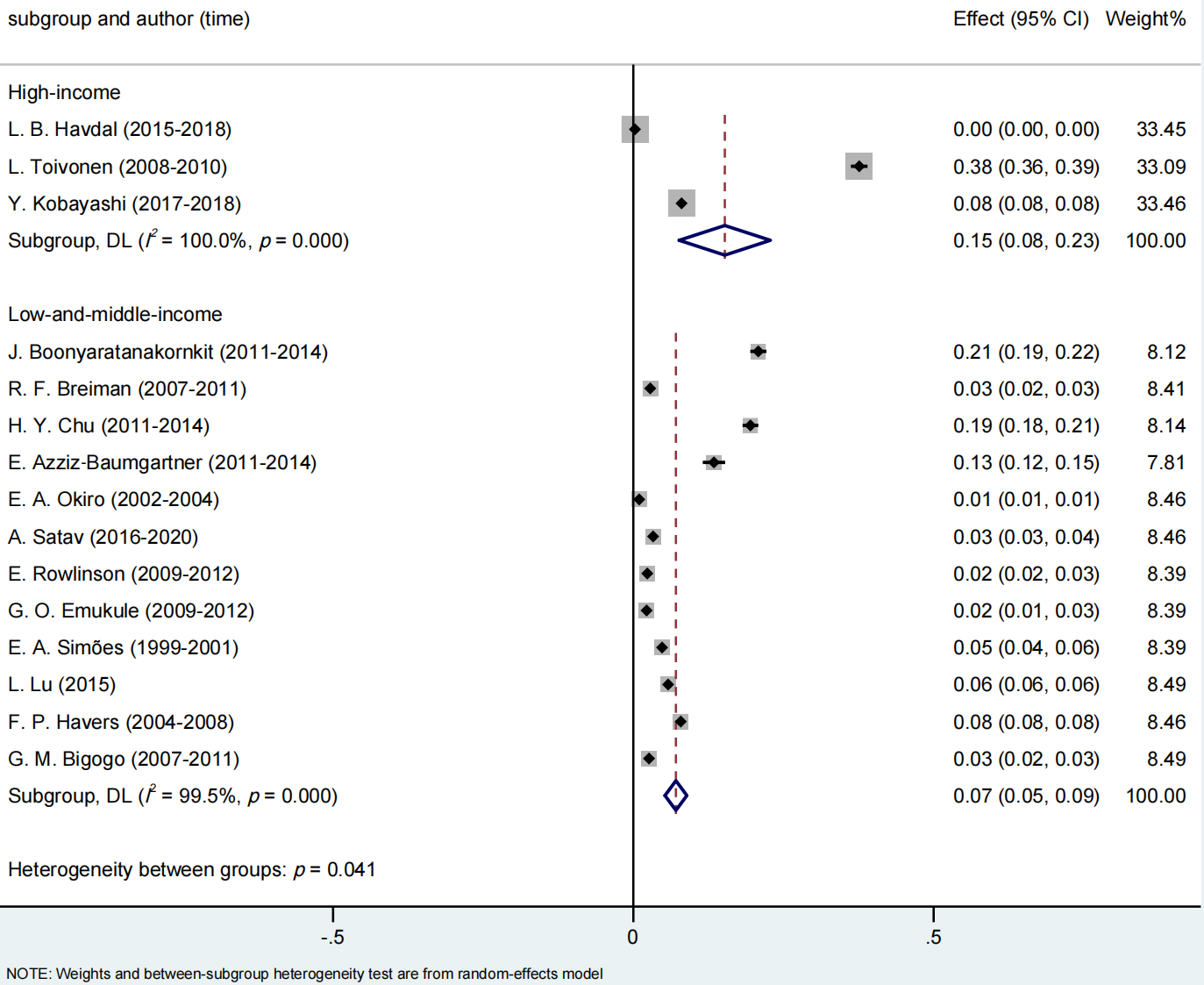 |
| **Income levels** |
| 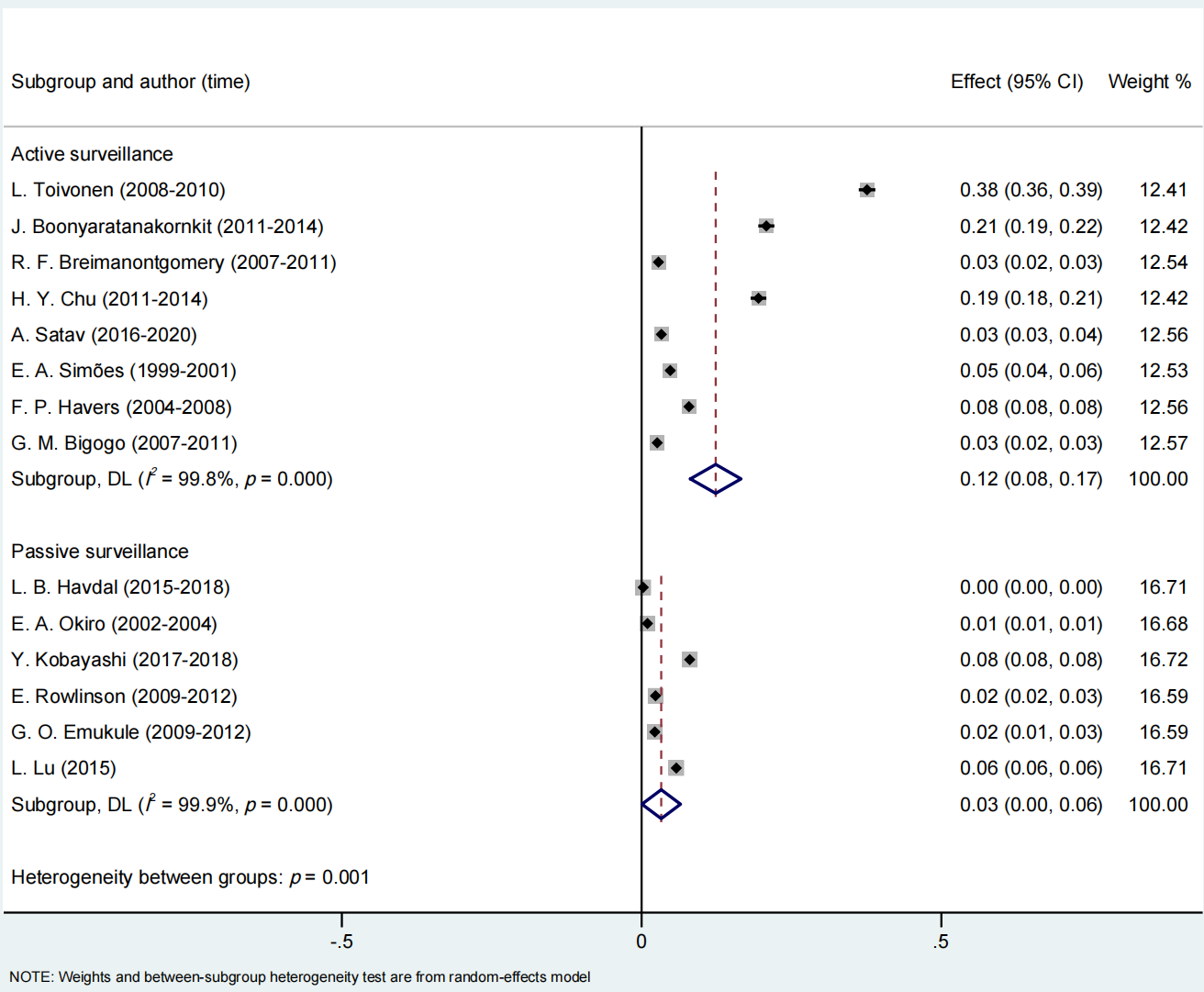 |
| **Surveillance types** |
| 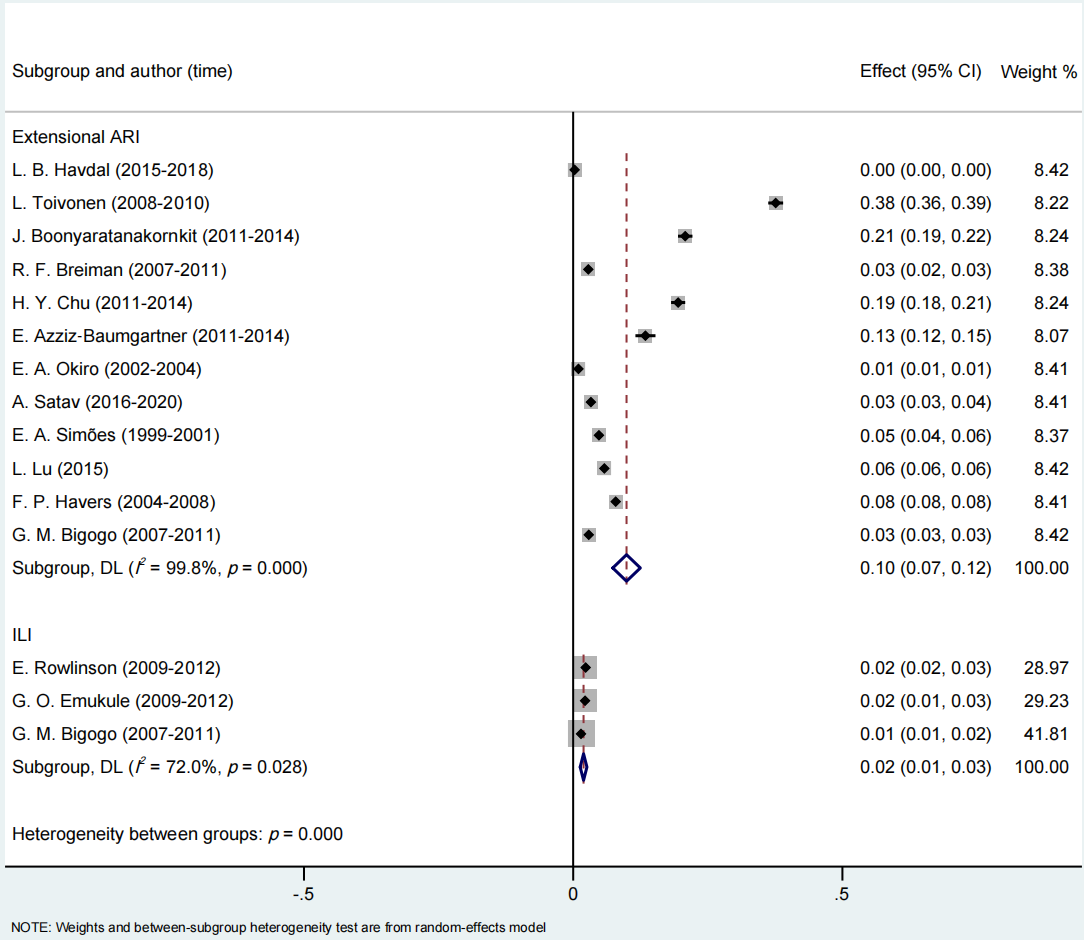 |
| **Case definition** |

**Supplementary figure 1. Subgroup analysis of RSV-related incidence among children ≤ 5 years old.** ARI = acute respiratory tract infection; ILI = influenza-like illness; CI = confidence interval; RSV = respiratory syncytial virus; 0-1 years old included children aged one-year-old and younger, 0-2 years old included children aged two-years-old and younger, 2-5 years old included three- , four- , and five-years-old children; Effect = RSV-related incidence per children per year.

| 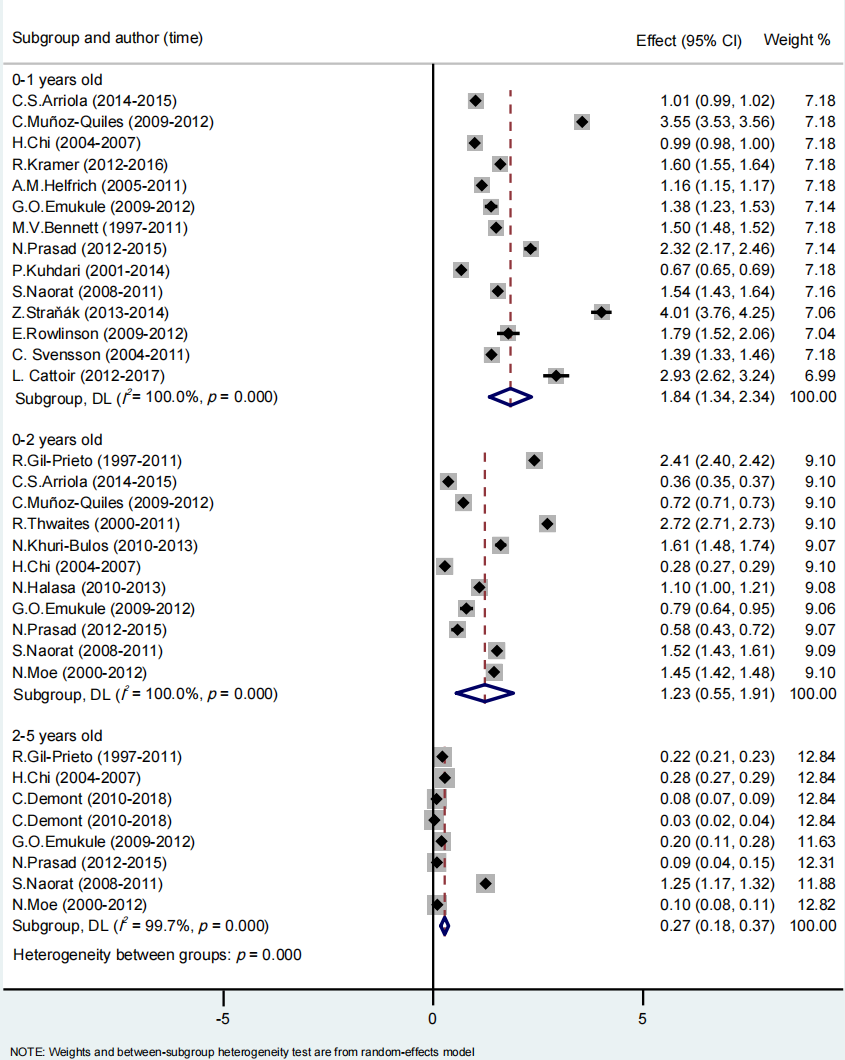 |
| --- |
| **Ages** |
| 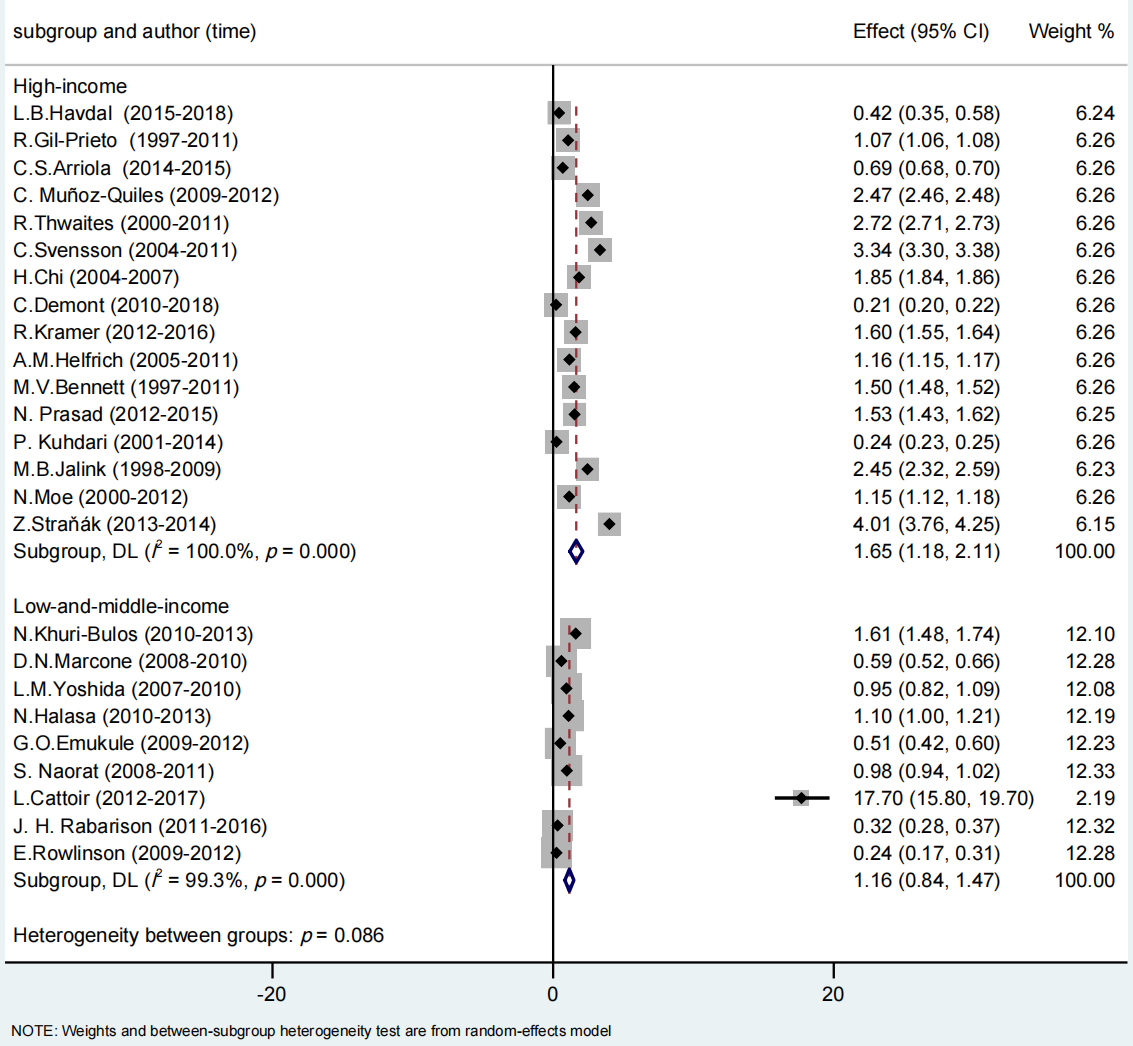 |
| **Income levels** |

**Supplementary figure 2. Subgroup analysis of RSV-related** **hospitalization rate among children** **≤ 5 years old.** CI = confidence interval; RSV = respiratory syncytial virus; 0-1 years old included children aged one-year-old and younger, 0-2 years old included children aged two-years-old and younger, 2-5 years old included three- , four- , and five-years-old children; Effect = RSV-related hospitalization rate per 100 children per year.

| 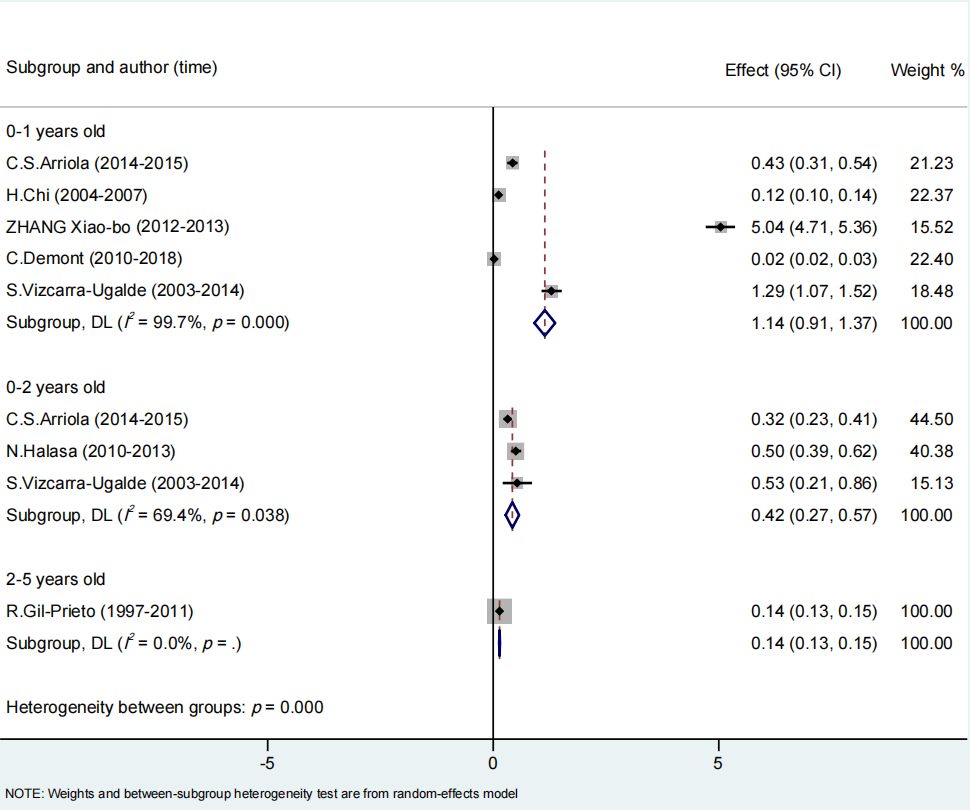 |
| --- |
| **Ages** |
| 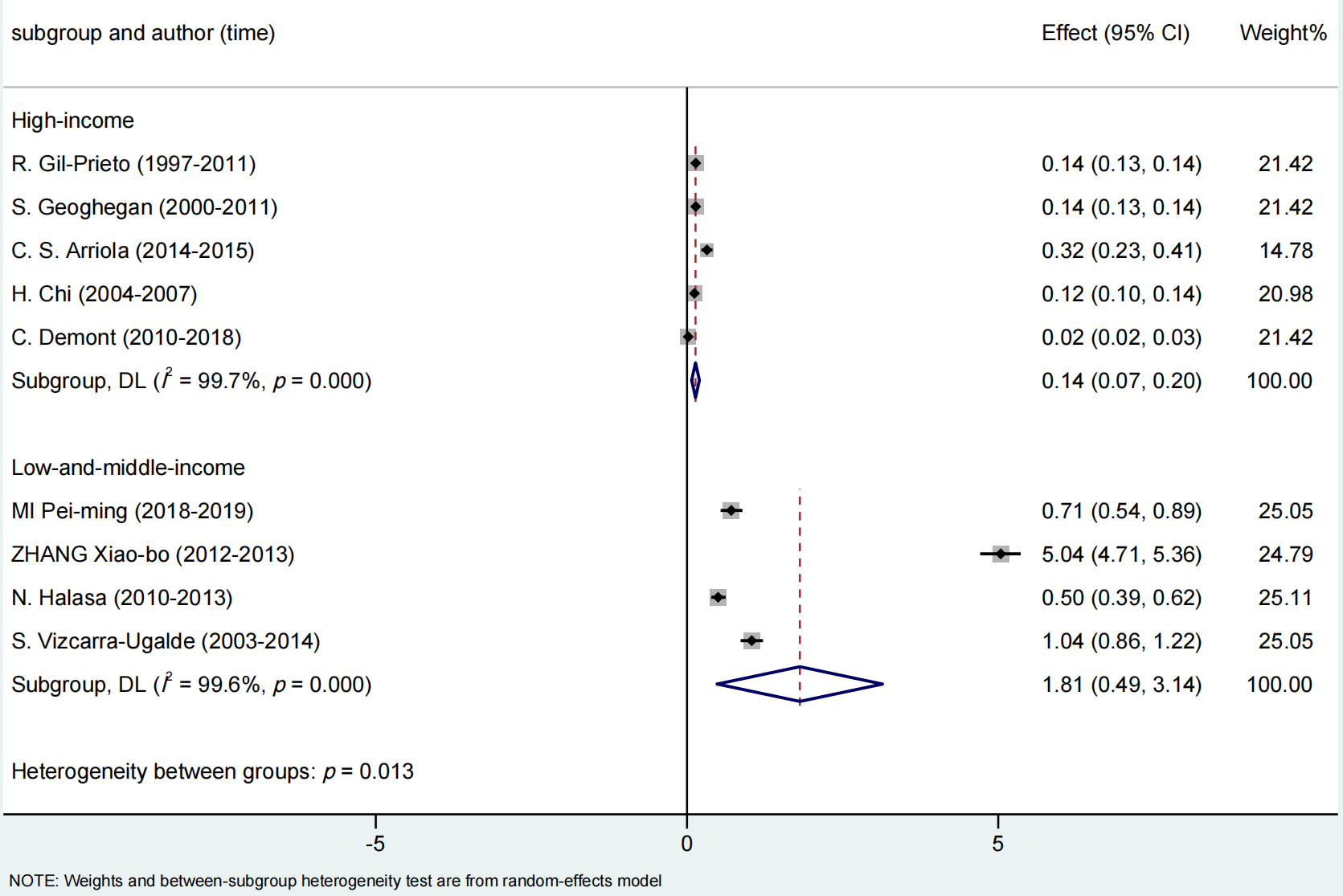 |
| **Income levels** |
| 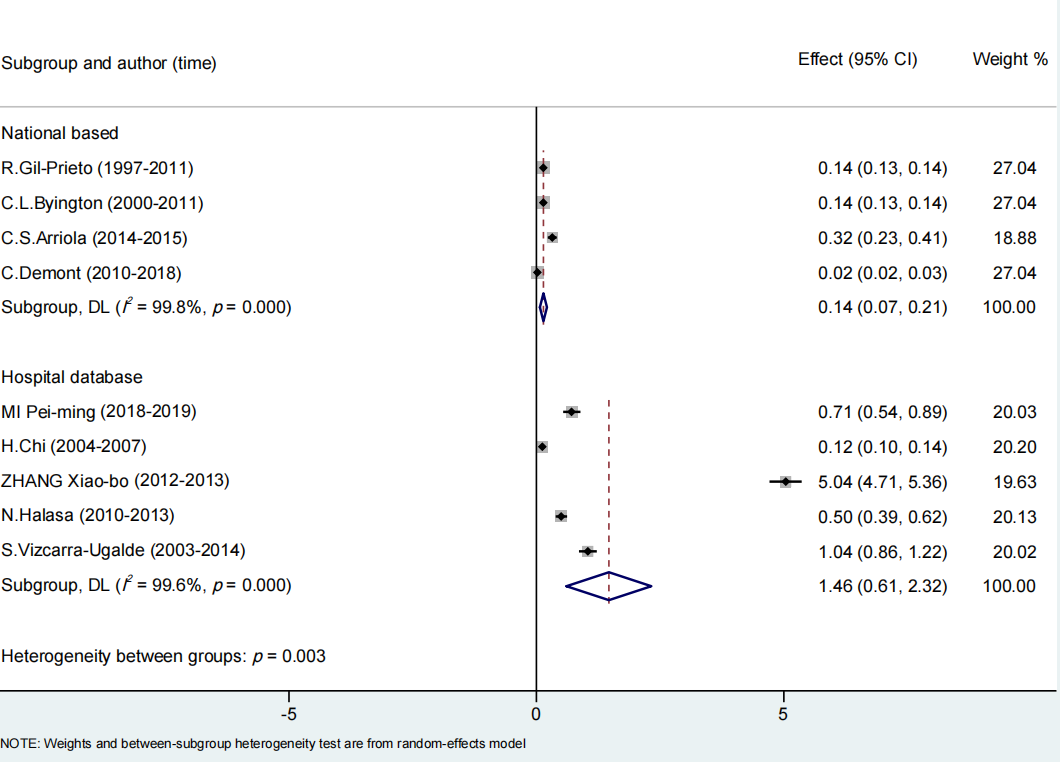 |
| **Data source** |

**Supplementary figure 3. Subgroup analysis of RSV-****related in-hospital mortality rate among children ≤ 5 years old.** CI = confidence interval; RSV = respiratory syncytial virus; 0-1 years old included children aged one-year-old and younger, 0-2 years old included children aged two-years-old and younger, 2-5 years old included three- , four- , and five-years-old children; Effect = RSV-related in-hospital mortality rate per 100 children per year.

| 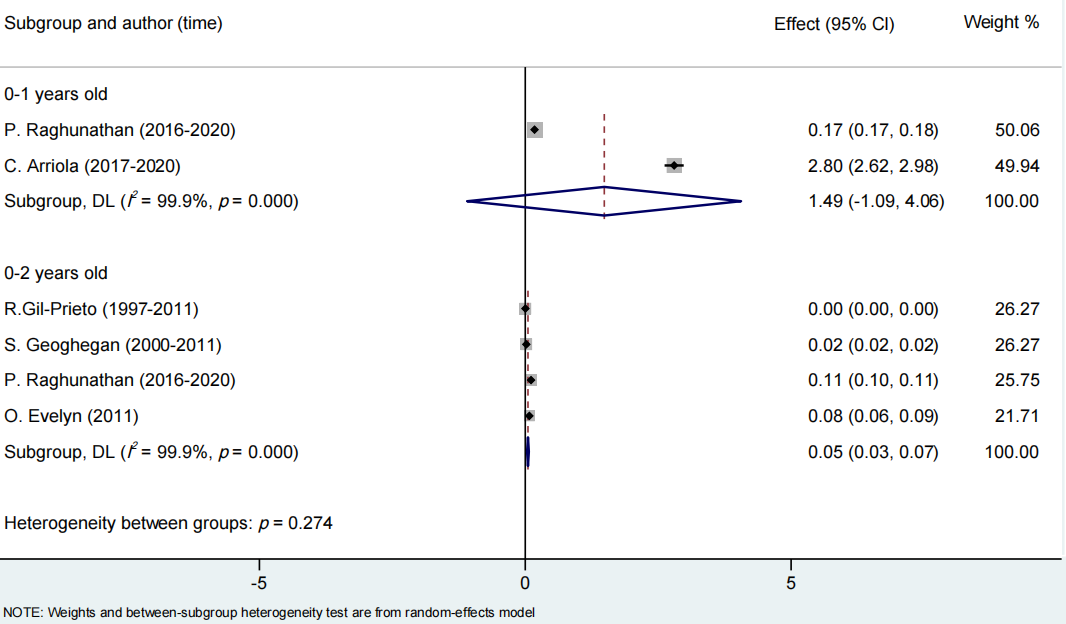 |
| --- |
| **Ages** |
| 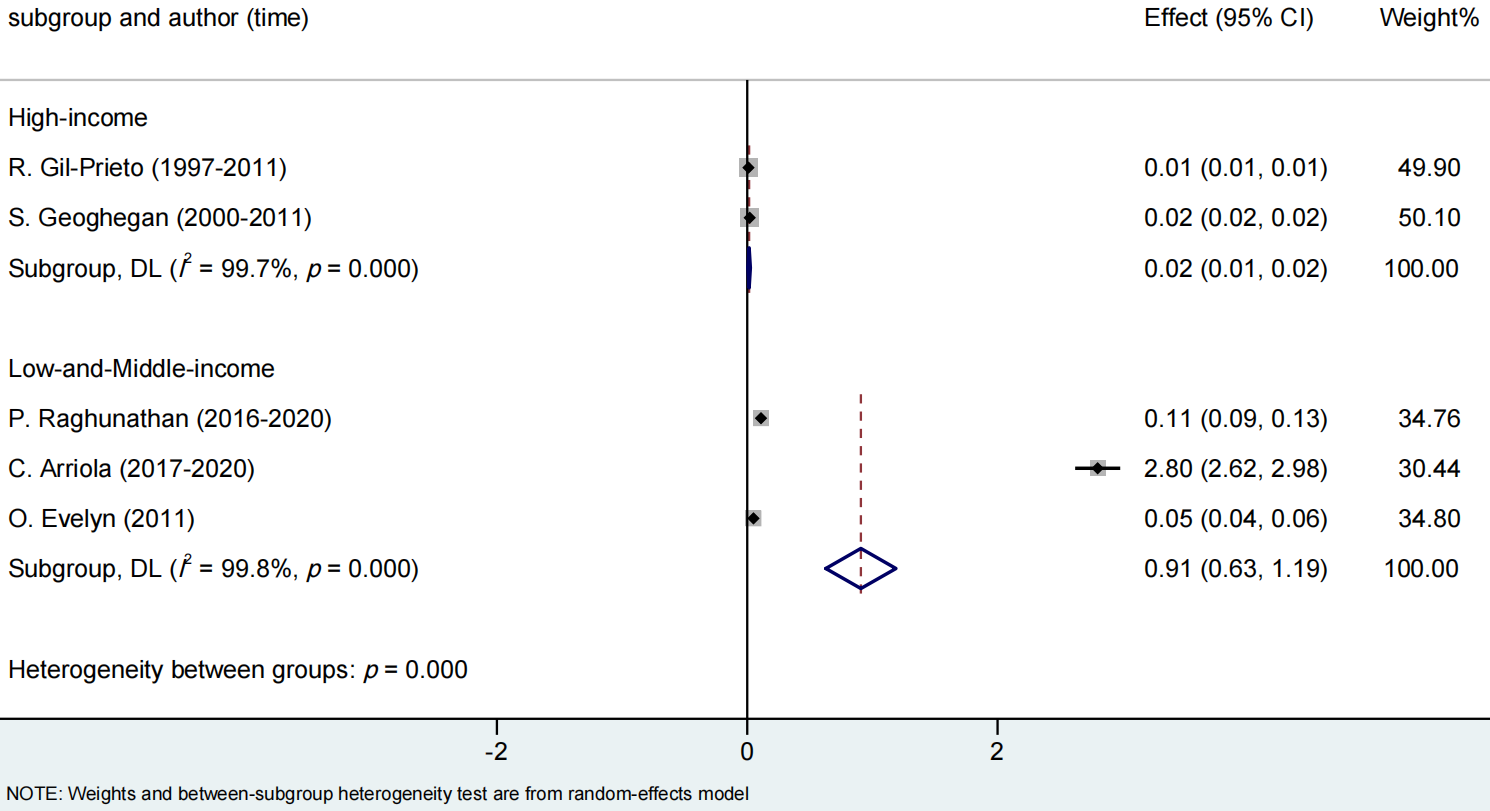 |
| **Income levels** |

**Supplementary figure 4. Subgroup analysis of RSV-related overall mortality rate among children ≤5 years old.** CI = confidence interval; RSV = respiratory syncytial virus; 0-1 years old included children aged one-year-old and younger, 0-2 years old included children aged two-years-old and younger, 2-5 years old included three- , four- , and five-years-old children; Effect = RSV-related overall mortality rate per 100 children per year.

| 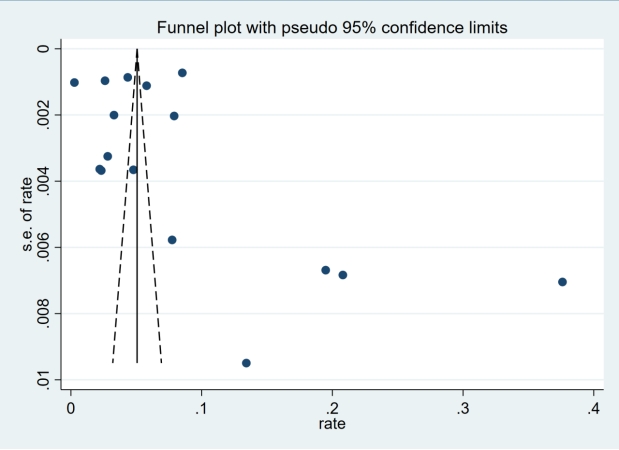 | 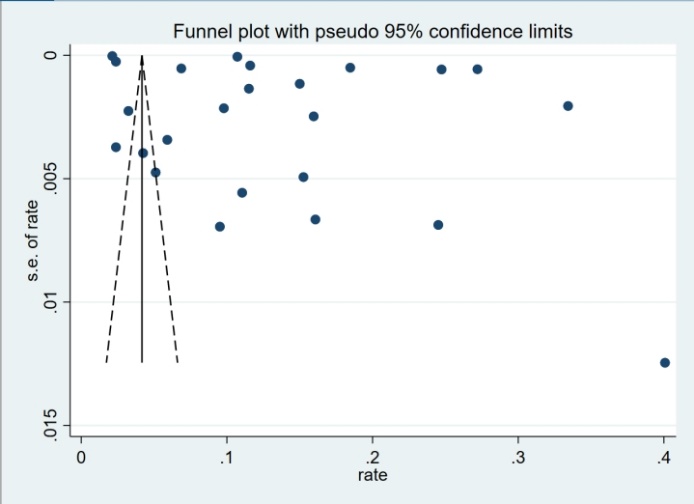 |
| --- | --- |
| **Incidence (**Egger’s test: *P*=0.453**)** | **Hospitalization rate (**Egger’s test: *P*=0.132**)** |
| 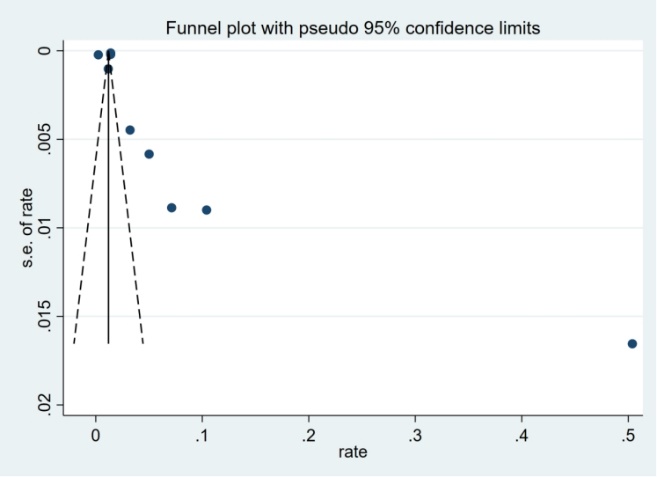 | 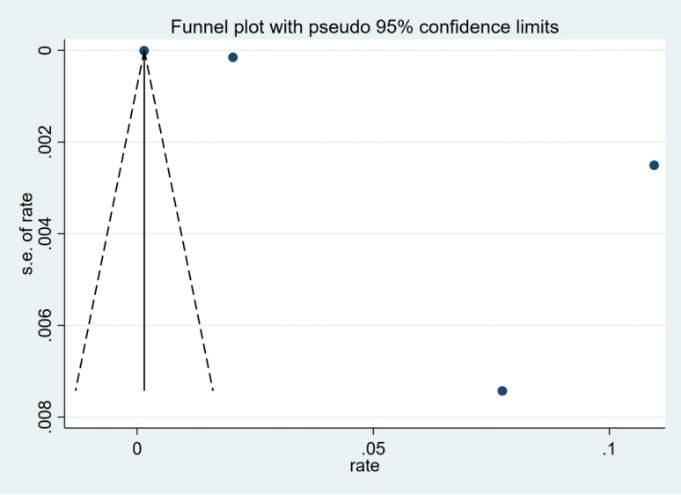 |
| **Hospital facility rate (**Egger’s test: *P*=0.417**)** | **Mortality (**Egger’s test: *P*=0.088**)** |

**Supplementary figure 5. Publication bias analyzed by funnel plot.**

| 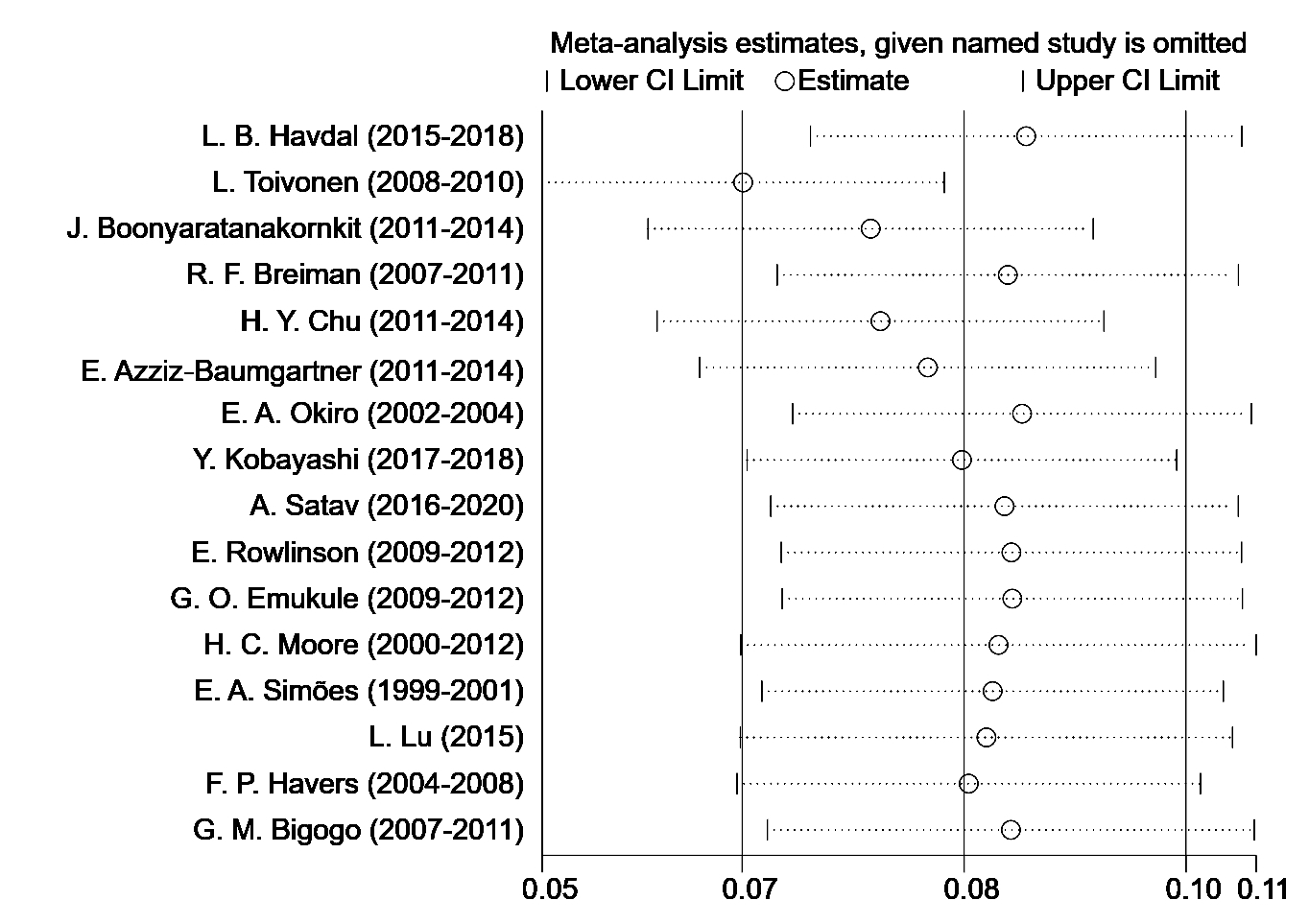 | | 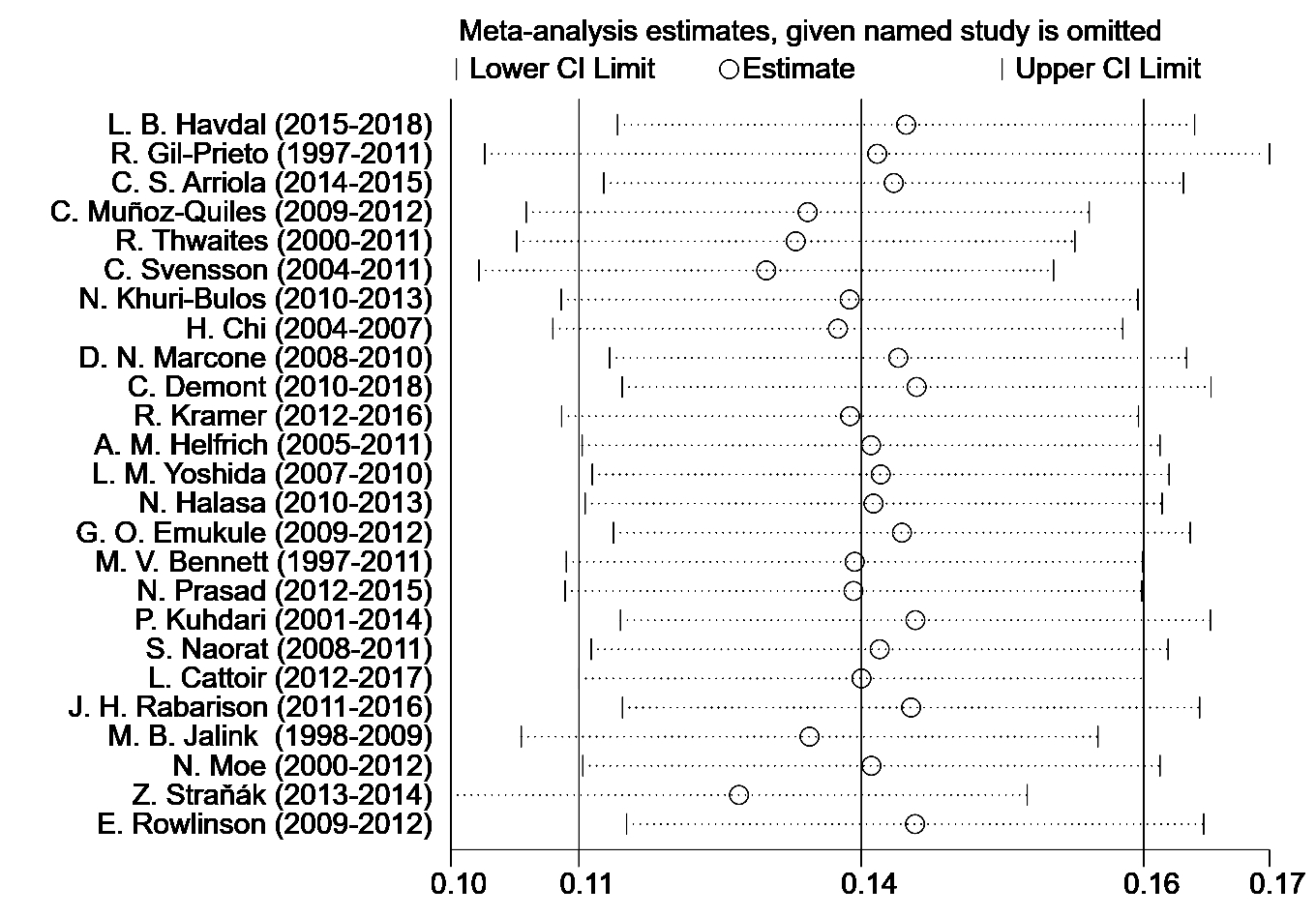 |
| --- | --- | --- |
| **Incidence rate** | | **Hospitalization rate** |
| 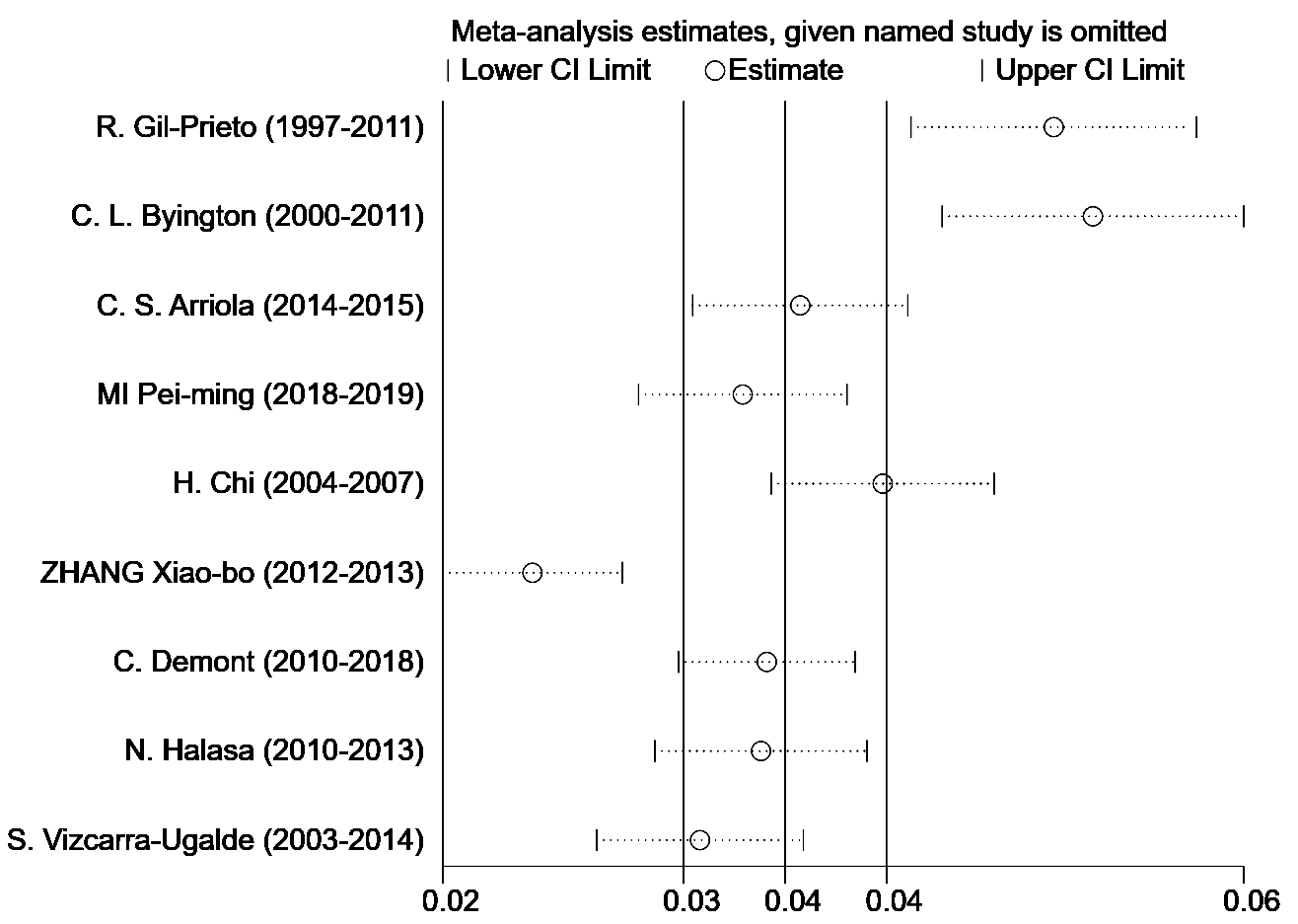 | | 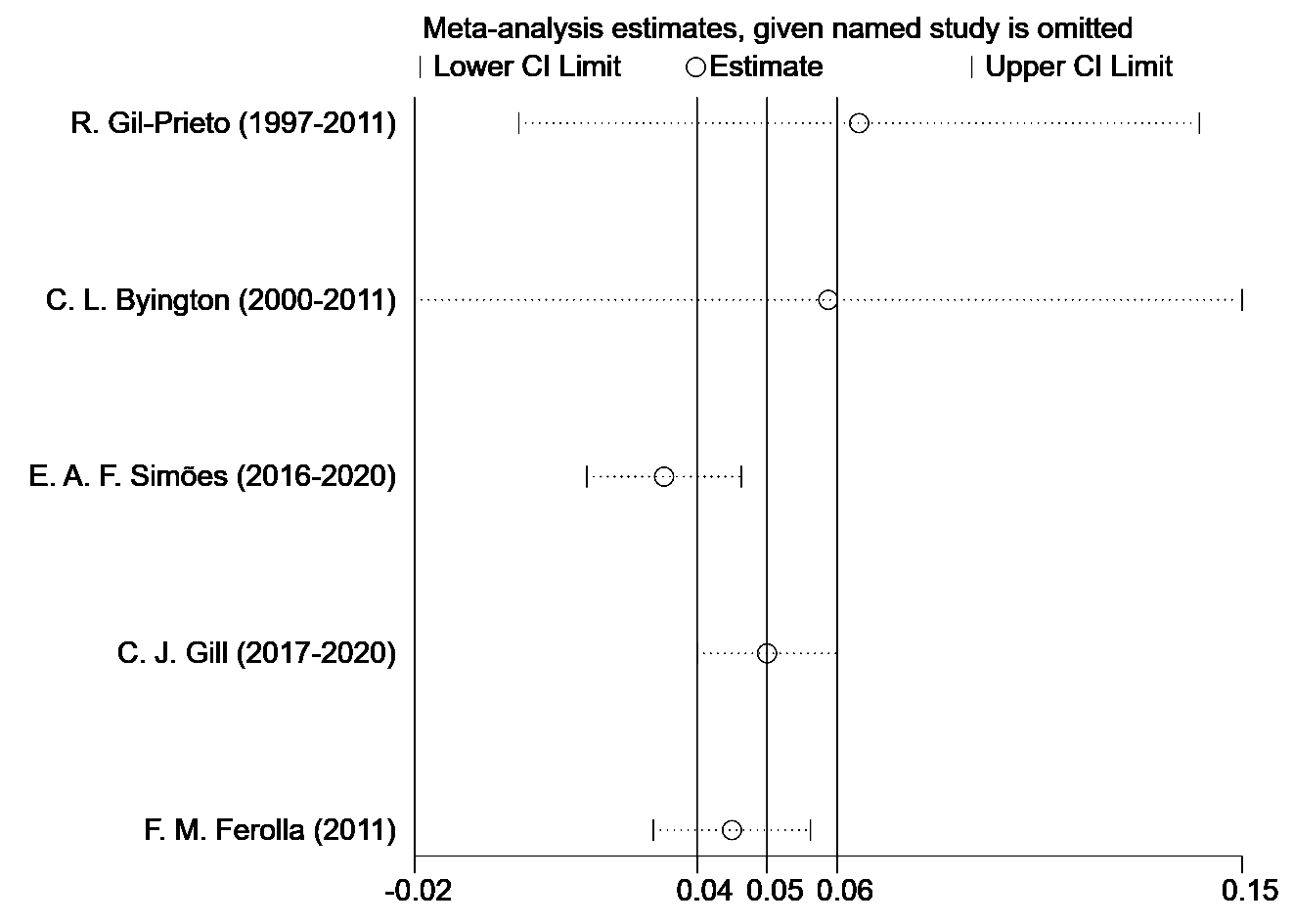 |
| **In-hospital mortality rate** | **Overall mortality rate** | |

**Supplementary Figure 6. Sensitivity analysis.**

**Supplementary table 7.** **Quality scoring criteria for observational study studies.**

The tool contains 11 items. the answer “yes” is scored one point and “no” or “not clear” is scored zero point. Moreover, studies were classified as “high quality” (8–11), “moderate quality” (4–7), and “low quality” (0–3) based on their total scores.

| **No** | | **The first author** | **Study Period** | **①** | **②** | **③** | **④** | **⑤** | **⑥** | **⑦** | **⑧** | **⑨** | **⑩** | **⑪** | **QA score** | **Outcomes** |
| --- | --- | --- | --- | --- | --- | --- | --- | --- | --- | --- | --- | --- | --- | --- | --- | --- |
| 1 | | L. B. Havdal | 2015-2018 | 1 | 1 | 1 | 1 | 1 | 1 | 1 | 1 | 1 | 0 | 1 | 10 | a, b |
| 2 | | L. Toivonen | 2008-2010 | 1 | 1 | 1 | 1 | 1 | 1 | 0 | 1 | 0 | 0 | 1 | 8 | a |
| 3 | | J. Boonyaratanakornkit | 2011-2014 | 1 | 1 | 1 | 1 | 1 | 1 | 0 | 1 | 0 | 0 | 1 | 8 | a |
| 4 | | R. F. Breiman | 2007-2011 | 1 | 1 | 1 | 1 | 1 | 1 | 0 | 1 | 0 | 0 | 1 | 8 | a |
| 5 | | H. Y. Chu | 2011-2014 | 1 | 1 | 1 | 1 | 1 | 1 | 1 | 1 | 1 | 0 | 1 | 10 | a |
| 6 | | E. Azziz‐Baumgartner | 2011-2014 | 1 | 1 | 1 | 1 | 1 | 1 | 0 | 1 | 0 | 0 | 1 | 8 | a |
| 7 | | E. A. Okiro | 2002-2004 | 1 | 1 | 1 | 1 | 1 | 1 | 1 | 1 | 0 | 1 | 1 | 10 | a |
| 8 | | Y. Kobayashi | 2017-2018 | 1 | 0 | 1 | 0 | 1 | 1 | 0 | 1 | 0 | 1 | 1 | 7 | a |
| 9 | | A. Satav | 2016-2020 | 1 | 1 | 1 | 1 | 1 | 1 | 0 | 1 | 0 | 0 | 0 | 7 | a |
| 10 | | E. Rowlinson | 2009-2012 | 1 | 1 | 1 | 1 | 1 | 1 | 1 | 1 | 0 | 0 | 0 | 8 | a, b |
| 11 | | G. O. Emukule | 2009-2012 | 1 | 1 | 1 | 0 | 1 | 1 | 1 | 1 | 0 | 1 | 1 | 9 | a, b |
| 12 | | H. C. Moore | 2000-2012 | 1 | 1 | 1 | 1 | 0 | 0 | 1 | 1 | 0 | 1 | 1 | 8 | a, b |
| 13 | | E. A. Simões | 1999-2001 | 1 | 1 | 1 | 0 | 1 | 1 | 0 | 1 | 0 | 1 | 1 | 8 | a |
| 14 | | L. Lu | 2015 | 1 | 1 | 1 | 1 | 0 | 1 | 0 | 1 | 0 | 1 | 1 | 8 | a |
| 15 | | F. P. Havers | 2004-2008 | 1 | 1 | 1 | 1 | 0 | 1 | 0 | 1 | 1 | 1 | 1 | 9 | a |
| 16 | | G. M. Bigogo | 2007-2011 | 1 | 1 | 1 | 0 | 1 | 1 | 1 | 1 | 0 | 1 | 1 | 9 | a |
| 17 | | R. Gil-Prieto | 1997-2011 | 1 | 1 | 1 | 1 | 0 | 1 | 0 | 1 | 0 | 1 | 0 | 7 | b, c, d |
| 18 | | C. S. Arriola | 2014-2015 | 1 | 1 | 1 | 1 | 1 | 1 | 1 | 1 | 0 | 1 | 1 | 10 | b, c |
| 19 | | C. Muñoz-Quiles | 2009-2012 | 1 | 1 | 1 | 1 | 0 | 1 | 1 | 1 | 0 | 1 | 1 | 9 | b |
| 20 | | R. Thwaites | 2000-2011 | 1 | 1 | 1 | 1 | 0 | 1 | 0 | 1 | 0 | 1 | 1 | 8 | b |
| 21 | | C. Svensson | 2004-2011 | 1 | 1 | 1 | 0 | 0 | 1 | 1 | 1 | 0 | 1 | 1 | 8 | b |
| 22 | | N. Khuri-Bulos | 2010-2013 | 1 | 1 | 1 | 0 | 0 | 1 | 0 | 1 | 1 | 1 | 1 | 8 | b |
| 23 | | H. Chi | 2004-2007 | 1 | 1 | 1 | 1 | 0 | 0 | 0 | 1 | 0 | 1 | 1 | 7 | b, c |
| 24 | | D. N. Marcone | 2008-2010 | 1 | 1 | 1 | 0 | 0 | 1 | 1 | 1 | 0 | 1 | 1 | 8 | b |
| 25 | | C. Demont | 2010-2018 | 1 | 1 | 1 | 1 | 0 | 1 | 0 | 1 | 0 | 1 | 1 | 8 | b, c |
| 26 | | R. Kramer | 2012-2016 | 1 | 1 | 1 | 0 | 0 | 1 | 0 | 1 | 0 | 1 | 1 | 7 | b |
| 27 | | A. M. Helfrich | 2005-2011 | 1 | 1 | 1 | 0 | 0 | 1 | 1 | 1 | 0 | 1 | 1 | 8 | b |
| 28 | | L. M. Yoshida | 2007-2010 | 1 | 1 | 1 | 1 | 1 | 1 | 1 | 1 | 0 | 1 | 1 | 10 | b |
| 29 | | Z. Straňák | 2013-2014 | 1 | 1 | 1 | 0 | 1 | 1 | 1 | 1 | 0 | 1 | 1 | 9 | b |
| 30 | | N. Halasa | 2010-2013 | 1 | 1 | 1 | 0 | 1 | 1 | 0 | 1 | 0 | 1 | 1 | 8 | b, c |
| 31 | | M. V. Bennett | 1997-2011 | 1 | 1 | 1 | 1 | 1 | 1 | 0 | 1 | 0 | 1 | 1 | 9 | b |
| 32 | | N. Prasad | 2012-2015 | 1 | 1 | 1 | 0 | 0 | 1 | 1 | 1 | 0 | 1 | 1 | 8 | b |
| 33 | | P. Kuhdari | 2001-2014 | 1 | 1 | 1 | 0 | 0 | 1 | 0 | 1 | 0 | 0 | 1 | 6 | b |
| 34 | | S. Naorat | 2008-2011 | 1 | 1 | 1 | 1 | 1 | 1 | 0 | 1 | 1 | 0 | 1 | 9 | b |
| 35 | | S. J. Quevedo Teruel | 2011-2016 | 1 | 1 | 1 | 0 | 0 | 1 | 0 | 1 | 1 | 1 | 1 | 8 | b |
| 36 | | M. B. Jalink | 1998-2009 | 1 | 1 | 1 | 0 | 1 | 1 | 1 | 1 | 0 | 1 | 1 | 9 | b |
| 37 | | L. Cattoir | 2012-2017 | 1 | 1 | 1 | 0 | 0 | 1 | 0 | 1 | 0 | 1 | 1 | 7 | b |
| 38 | C. L. Byington | | 2000-2011 | 1 | 1 | 1 | 1 | 0 | 1 | 0 | 1 | 0 | 1 | 1 | 8 | c, d |
| 39 | MI Pei-ming | | 2018-2019 | 1 | 1 | 1 | 0 | 0 | 1 | 0 | 1 | 0 | 1 | 1 | 7 | c |
| 40 | ZHANG Xiao-bo | | 2012-2013 | 1 | 1 | 1 | 0 | 0 | 1 | 0 | 1 | 0 | 1 | 1 | 7 | c |
| 41 | S. Vizcarra-Ugalde | | 2003-2014 | 1 | 1 | 1 | 0 | 0 | 1 | 0 | 1 | 0 | 1 | 1 | 7 | c |
| 42 | P. Raghunathan | | 2016-2020 | 1 | 1 | 1 | 1 | 1 | 1 | 0 | 1 | 0 | 1 | 1 | 9 | d |
| 43 | C. Arriola | | 2017-2020 | 1 | 1 | 1 | 0 | 1 | 1 | 1 | 1 | 1 | 1 | 1 | 10 | d |
| 44 | O. Evelyn | | 2011 | 1 | 1 | 1 | 0 | 1 | 1 | 0 | 1 | 0 | 1 | 1 | 8 | d |

a: Incidence rate; b: Hospitalization rate; c: hospital facility rate; d: Mortality.

①Define the source of information (survey, record review;

②List inclusion and exclusion criteria for exposed and unexposed subjects (cases and control) or refer to previous publications;

③Indicate time period used for identifying patients;

④Indicate whether or not subjects were consecutive if not population-based;

⑤Indicate if evaluators of subjective components of study were masked to other aspects of the status of the participants;

⑥Describe any assessments undertaken for quality assurance purposes (e. g. test/retest of primary outcome measurements);

⑦Explain any patient exclusions from analysis;

⑧Describe how confounding was assessed and/or controlled;

⑨If applicable, explain how missing data were handled in the analysis;

⑩Summarize patient response rates and completeness of data collection;

⑪Clarify what follow-up, if any, was expected and the percentage of patients for which incomplete data or follow-up was obtained;

**Supplementary table 8. Comparation of RSV-related disease burden in different studies.**

| **Subgroups** | **Our study*** | **Li et al.** | **Shi et al.** |
| --- | --- | --- | --- |
|  | Synthetic rate from 2010 to 2022 by meta-analysis | Estimated rate in 2019 by modeling study | Estimated rate in 2015 by modeling study |
| **Total** | | |  |
| Incidence rate | 9.0 (7.0-11.0) † | 4.9 (3.7-6.6) | 3.6 (1.7-7.3) |
| hospitalization rate | 1.7 (1.3-2.1) | 0.5 (0.4-0.7) | 0.5 (0.4-0.7) |
| in-hospital mortality rate | 0.5 (0.4-0.5) | 0.7 (0.5-1.1) | 1.9 (1.7-2.0) |
| overall mortality rate | 0.05 (0.04-0.06) | 0.2 (1.6-2.4) | - |
| **Income levels** | | | |
| **Incidence rate** |  |  |  |
| High-income | 15.0 (8.0-23.0) | 2.4 (1.4-4.3) | 3.6 (1.7-7.6) |
| Low-and-middle-income | 7.0 (5.0-9.0) | 5.2 (3.1-9.1) | 7.3 (5.0-12.7) |
| **Hospitalization rate** |  |  |  |
| High-income | 1.7 (1.2-2.1) | 0.6 (0.5-0.8) | 0.5(0.4-0.7) |
| Low-and-middle-income | 1.2 (0.8-1.5) | 0.5 (0.3-0.9) | 0.4 (0.3-0.6) |
| **In-hospital mortality rate** |  |  |  |
| High-income | 0.1 (0.1-0.2) | 0.1 (0.1–0.2) | 0.9 (0.2-1.2) |
| Low-and-middle-income | 1.8 (0.5-3.1) | 0.8 (0.6–1.2) | 2.5 (2.1-3.4) |
| **Overall mortality rate** |  |  |  |
| High-income | 0.02 (0.01-0.02) | 0.1 (0.1-0.2) | - |
| Low-and-middle-income | 0.9 (0.6-1.2) | 0.2 (0.1-0.3) | - |

*To include more studies and expand the age range appropriately, the age structure used in our study were children ≤5 years rather than under 5 years as normally used; †Per 100 children per year.

**Outliers’ analysis：**

The included studies had some obviously outliers and considerable heterogeneity owing to the differences in income level, age groups, surveillance types, case ascertainment standards, the diagnostic assays used to identify RSV, and the disparity in access to hospital care across studies. However, we did not exclude those studies because they met the inclusion criteria. We calculated the pooled rate and used a random-effects model to synthesize the disease burden, which allows for some outliers and heterogeneity. The sensitivity analyses also confirmed the stability of the result. In addition, it is the aim of our study to explore the influencing factors of heterogeneity, thus avoiding a lack of comparability between studies in the future. We conducted a supplementary analysis of the studies that constituted the outliers in this section.

The studies of Havdal et al. and Toivonen et al. in Figure 2A, Cattoir et al in Fig 2B, Zhang et al Fig 2C and Gill et al Fig 2D, all look to be potential outliers. The study conducted by Havdal et al. was a passive surveillance study, and they included 1–5 years old children referred to hospital with fever, which may under-estimate the number of RSV-infected patients. On the other side, if the same patient had both in- and outpatient contacts during the 21-day period, only the inpatient contact was used in the study, which may further under-estimate the RSV-related incidence. The study conducted by Toivonen et al. was an active community surveillance study, including intensive follow-up with diaries, study clinic visits, home sampling, and a review of medical records, which may lead to a high RSV-related incidence rate. The rate of RSV-related hospitalization was as high as 17.7 per 100 children per year in the study conducted by L. Cattoir et al. The patients in this study were children aged 0–4 years with an RSV-related severe acute respiratory infection. Compared with patients with an acute respiratory tract infection, patients with a severe acute respiratory infection are more likely to be hospitalized, so the hospitalization rate is higher than that of ARI patients. The RSV-related in-hospital mortality rate obtained by Zhang et al. was higher than that of other studies, presumably because the study was conducted in a middle-income country, and the population included in the study was 0–1-year-old children, which may jointly lead to a high in-hospital mortality rate. The RSV-related overall mortality rate obtained by Arriola et al. was higher than other studies, presumably because the study was carried out in a low-income country and the age of the included population was 0–6 months. Therefore, the combined effect of economics and age may be the main reason for the high mortality rate in this study.

**Reference：**

1. Teirlinck AC, Broberg EK, Berg AS, et al. Recommendations for respiratory syncytial virus surveillance at the national level. *European Respiratory Journal.* 2021;58(3).

2. Nair H, Nokes DJ, Gessner BD, et al. Global burden of acute lower respiratory infections due to respiratory syncytial virus in young children: a systematic review and meta-analysis. *Lancet.* 2010;375(9725):1545-1555.

3. National Influenza Surveillance Programme (2017 edition). <http://www.nhc.gov.cn/jkj/s3577/201704/ed1498d9e64144738cc7f8db61a39506.shtml>.

4. World Bank income groups, 2021. 2023; <https://ourworldindata.org/grapher/world-banks-income-groups>. Accessed 04-13, 2023.

5. Data OWi. Human Development Index (HDI). <https://ourworldindata.org/human-development-index>.

6. Havdal LB, Bøås H, Bekkevold T, et al. The burden of respiratory syncytial virus in children under 5 years of age in Norway. *Journal of Infection.* 2022;84(2):205-215.

7. Toivonen L, Karppinen S, Schuez-Havupalo L, et al. Respiratory syncytial virus infections in children 0–24 months of age in the community. *Journal of Infection.* 2020;80(1):69-75.

8. Boonyaratanakornkit J, Englund JA, Magaret AS, et al. Primary and Repeated Respiratory Viral Infections Among Infants in Rural Nepal. *Journal of the Pediatric Infectious Diseases Society.* 2018;9(1):21-29.

9. Breiman RF, Cosmas L, Njenga MK, et al. Severe acute respiratory infection in children in a densely populated urban slum in Kenya, 2007–2011. *BMC Infectious Diseases.* 2015;15(1):95.

10. Chu HY, Katz J, Tielsch J, et al. Respiratory syncytial virus infection in infants in rural Nepal. *Journal of Infection.* 2016;73(2):145-154.

11. Azziz‐Baumgartner E, Bruno A, Daugherty M, et al. Incidence and seasonality of respiratory viruses among medically attended children with acute respiratory infections in an Ecuador birth cohort, 2011–2014. *Influenza and Other Respiratory Viruses.* 2022;16(1):24-33.

12. Okiro EA, Ngama M, Bett A, Nokes DJ. The incidence and clinical burden of respiratory syncytial virus disease identified through hospital outpatient presentations in Kenyan children. *PLoS One.* 2012;7(12):e52520.

13. Kobayashi Y, Togo K, Agosti Y, McLaughlin JM. Epidemiology of respiratory syncytial virus in Japan: A nationwide claims database analysis. *Pediatrics International.* 2022;64(1):e14957.

14. Satav A, Crow R, Potdar V, et al. The burden of respiratory syncytial virus in children under 2 years of age in a rural community in Maharashtra, India. *Clinical Infectious Diseases.* 2021;73(Supplement_3):S238-S247.

15. Rowlinson E, Dueger E, Taylor T, et al. Incidence and Clinical Features of Respiratory Syncytial Virus Infections in a Population-Based Surveillance Site in the Nile Delta Region. *The Journal of Infectious Diseases.* 2013;208(suppl_3):S189-S196.

16. Emukule GO, Khagayi S, McMorrow ML, et al. The burden of influenza and RSV among inpatients and outpatients in rural western Kenya, 2009–2012. *PloS one.* 2014;9(8):e105543.

17. Simões EA, Chirikov V, Botteman M, Kwon Y, Kuznik A. Long-term assessment of healthcare utilization 5 years after respiratory syncytial virus infection in US infants. *The Journal of Infectious Diseases.* 2020;221(8):1256-1270.

18. Liu L, Oza S, Hogan D, et al. Global, regional, and national causes of under-5 mortality in 2000-15: an updated systematic analysis with implications for the Sustainable Development Goals. *Lancet.* 2016;388(10063):3027-3035.

19. Havers FP, Fry AM, Goswami D, et al. Population-based incidence of childhood pneumonia associated with viral infections in Bangladesh. *The Pediatric infectious disease journal.* 2019;38(4):344-350.

20. Bigogo GM, Breiman RF, Feikin DR, et al. Epidemiology of Respiratory Syncytial Virus Infection in Rural and Urban Kenya. *The Journal of Infectious Diseases.* 2013;208(suppl_3):S207-S216.

21. Gil-Prieto R, Gonzalez-Escalada A, Marín-García P, Gallardo-Pino C, Gil-de-Miguel A. Respiratory Syncytial Virus Bronchiolitis in Children up to 5 Years of Age in Spain: Epidemiology and Comorbidities: An Observational Study. *Medicine (Baltimore).* 2015;94(21):e831.

22. Arriola CS, Kim L, Langley G, et al. Estimated Burden of Community-Onset Respiratory Syncytial Virus–Associated Hospitalizations Among Children Aged &lt;2 Years in the United States, 2014–15. *Journal of the Pediatric Infectious Diseases Society.* 2019;9(5):587-595.

23. Muñoz-Quiles C, López-Lacort M, Úbeda-Sansano I, et al. Population-based analysis of bronchiolitis epidemiology in Valencia, Spain. *The Pediatric infectious disease journal.* 2016;35(3):275-280.

24. Thwaites R, Buchan S, Fullarton J, et al. Clinical burden of severe respiratory syncytial virus infection during the first 2 years of life in children born between 2000 and 2011 in Scotland. *European Journal of Pediatrics.* 2020;179(5):791-799.

25. Svensson C, Berg K, Sigurs N, Trollfors B. Incidence, risk factors and hospital burden in children under five years of age hospitalised with respiratory syncytial virus infections. *Acta paediatrica.* 2015;104(9):922-926.

26. Khuri-Bulos N, Lawrence L, Piya B, et al. Severe outcomes associated with respiratory viruses in newborns and infants: a prospective viral surveillance study in Jordan. *BMJ Open.* 2018;8(5):e021898.

27. Chi H, Chang IS, Tsai F-Y, et al. Epidemiological Study of Hospitalization Associated With Respiratory Syncytial Virus Infection in Taiwanese Children Between 2004 and 2007. *Journal of the Formosan Medical Association.* 2011;110(6):388-396.

28. Marcone DN, Durand LO, Azziz-Baumgartner E, et al. Incidence of viral respiratory infections in a prospective cohort of outpatient and hospitalized children aged ≤5 years and its associated cost in Buenos Aires, Argentina. *BMC Infectious Diseases.* 2015;15(1):447.

29. Demont C, Petrica N, Bardoulat I, et al. Economic and disease burden of RSV-associated hospitalizations in young children in France, from 2010 through 2018. *BMC Infectious Diseases.* 2021;21(1):730.

30. Kramer R, Duclos A, Lina B, Casalegno J-S. Cost and burden of RSV related hospitalisation from 2012 to 2017 in the first year of life in Lyon, France. *Vaccine.* 2018;36(45):6591-6593.

31. Helfrich AM, Nylund CM, Eberly MD, Eide MB, Stagliano DR. Healthy Late-preterm infants born 33–36+6 weeks gestational age have higher risk for respiratory syncytial virus hospitalization. *Early Human Development.* 2015;91(9):541-546.

32. Yoshida L-M, Suzuki M, Nguyen HA, et al. Respiratory syncytial virus: co-infection and paediatric lower respiratory tract infections. *European Respiratory Journal.* 2013;42(2):461-469.

33. Halasa N, Williams J, Faouri S, et al. Natural history and epidemiology of respiratory syncytial virus infection in the Middle East: Hospital surveillance for children under age two in Jordan. *Vaccine.* 2015;33(47):6479-6487.

34. Bennett MV, McLaurin K, Ambrose C, Lee HC. Population-based trends and underlying risk factors for infant respiratory syncytial virus and bronchiolitis hospitalizations. *PLoS One.* 2018;13(10):e0205399.

35. Prasad N, Newbern EC, Trenholme AA, et al. Respiratory syncytial virus hospitalisations among young children: a data linkage study. *Epidemiology & Infection.* 2019;147.

36. Kuhdari P, Brosio F, Malaventura C, et al. Human respiratory syncytial virus and hospitalization in young children in Italy. *Italian Journal of Pediatrics.* 2018;44(1):1-7.

37. Naorat S, Chittaganpitch M, Thamthitiwat S, et al. Hospitalizations for acute lower respiratory tract infection due to respiratory syncytial virus in Thailand, 2008–2011. *The Journal of infectious diseases.* 2013;208(suppl_3):S238-S245.

38. Chavez D, Gonzales‐Armayo V, Mendoza E, et al. Estimation of influenza and respiratory syncytial virus hospitalizations using sentinel surveillance data—La Paz, Bolivia. 2012–2017. *Influenza and Other Respiratory Viruses.* 2019;13(5):477-483.

39. Rabarison JH, Tempia S, Harimanana A, et al. Burden and epidemiology of influenza‐and respiratory syncytial virus‐associated severe acute respiratory illness hospitalization in Madagascar, 2011‐2016. *Influenza and other respiratory viruses.* 2019;13(2):138-147.

40. Jalink MB, Langley JM, Dodds L, Andreou P. Severe respiratory syncytial virus infection in preterm infants and later onset of asthma. *The Pediatric Infectious Disease Journal.* 2019;38(11):1121-1125.

41. Moore HC, Lim FJ, Fathima P, et al. Assessing the Burden of Laboratory-Confirmed Respiratory Syncytial Virus Infection in a Population Cohort of Australian Children Through Record Linkage. *The Journal of Infectious Diseases.* 2020;222(1):92-101.

42. Straňák Z, Saliba E, Kosma P, et al. Predictors of RSV LRTI hospitalization in infants born at 33 to 35 weeks gestational age: a large multinational study (PONI). *PLoS One.* 2016;11(6):e0157446.

43. Byington CL, Wilkes J, Korgenski K, Sheng X. Respiratory syncytial virus-associated mortality in hospitalized infants and young children. *Pediatrics.* 2015;135(1):e24-31.

44. MI Pei-ming TM-f, HUANG Jia-yi, WEN Shang-mei, LIU Zhi-wei. Clinical analysis of 842 children with Respiratory Syncytial Virus pneumonia. *CHINA MODERN MEDICINE.* 2019;26(21):45-48.

45. Li-ling ZX-bWC-kLL-jJG-lWL-bSPXJQ. Clinical features and disease burden of acute low respiratory infection caused by respiratory syncytial virus in hospitalizedneonatesand infants. *Chin J Evid Based Pediatr.* 2014;9(01):45-48.

46. Vizcarra-Ugalde S, Rico-Hernández M, Monjarás-Ávila C, et al. Intensive care unit admission and death rates of infants admitted with respiratory syncytial virus lower respiratory tract infection in Mexico. *The Pediatric Infectious Disease Journal.* 2016;35(11):1199-1203.

47. Simões EAF, Dani V, Potdar V, et al. Mortality From Respiratory Syncytial Virus in Children Under 2 Years of Age: A Prospective Community Cohort Study in Rural Maharashtra, India. *Clinical Infectious Diseases.* 2021;73(Supplement_3):S193-S202.

48. Gill CJ, Mwananyanda L, MacLeod WB, et al. Infant deaths from respiratory syncytial virus in Lusaka, Zambia from the ZPRIME study: a 3-year, systematic, post-mortem surveillance project. *The Lancet Global Health.* 2022;10(2):e269-e277.

49. Ferolla FM, Hijano DR, Acosta PL, et al. Macronutrients during pregnancy and life-threatening respiratory syncytial virus infections in children. *American journal of respiratory and critical care medicine.* 2013;187(9):983-990.
